# Infrared Spectroscopy of Size‐Selected Hydrated Carbon Dioxide Radical Anions CO_2_^.−^(H_2_O)_*n*_ (*n*=2–61) in the C−O Stretch Region

**DOI:** 10.1002/chem.201901650

**Published:** 2019-07-01

**Authors:** Andreas Herburger, Milan Ončák, Chi‐Kit Siu, Ephrem G. Demissie, Jakob Heller, Wai Kit Tang, Martin K. Beyer

**Affiliations:** ^1^ Institut für Ionenphysik und Angewandte Physik Universität Innsbruck Technikerstraße 25 6020 Innsbruck Austria; ^2^ Department of Chemistry City University of Hong Kong 83 Tat Chee Avenue Kowloon Tong, Hong Kong SAR P. R. China

**Keywords:** ab initio calculations, carbon dioxide radical anions, clusters, infrared spectroscopy, mass spectrometry

## Abstract

Understanding the intrinsic properties of the hydrated carbon dioxide radical anions CO_2_^.−^(H_2_O)_*n*_ is relevant for electrochemical carbon dioxide functionalization. CO_2_^.−^(H_2_O)_*n*_ (*n*=2–61) is investigated by using infrared action spectroscopy in the 1150–2220 cm^−1^ region in an ICR (ion cyclotron resonance) cell cooled to *T=*80 K. The spectra show an absorption band around 1280 cm^−1^, which is assigned to the symmetric C−O stretching vibration *ν*_s_. It blueshifts with increasing cluster size, reaching the bulk value, within the experimental linewidth, for *n*=20. The antisymmetric C−O vibration *ν*_as_ is strongly coupled with the water bending mode *ν*_2_, causing a broad feature at approximately 1650 cm^−1^. For larger clusters, an additional broad and weak band appears above 1900 cm^−1^ similar to bulk water, which is assigned to a combination band of water bending and libration modes. Quantum chemical calculations provide insight into the interaction of CO_2_^.−^ with the hydrogen‐bonding network.

## Introduction

The carbon dioxide radical anion CO_2_^.−^ is a key intermediate in the electrochemical as well as catalytic activation of carbon dioxide, which is relevant for using CO_2_ as a C_1_ building block.^1^ The activation of CO_2_ by reductive electron transfer is well investigated, as reviewed recently by Weber.^2^, ^3^ With its charge, CO_2_^.−^ can be readily studied by mass spectrometry or in ion beams. Bare CO_2_^.−^ is a metastable species^4^, ^5^, ^6^ with a lifetime of tens of μs to ms.^3^ Upon solvation with molecules such as neutral CO_2_,^7^, ^8^ H_2_O,^9^ or both,^10^ however, it turns into a stable species. The carbon dioxide radical anion is highly reactive and forms C−C bonds in the reaction with allyl alcohol,^11^ methyl acrylate,^12^ and 3‐butyn‐1‐ol.^13^ Evidence for HOCO^.^ formation was found in the reactions with 3‐butyn‐1‐ol as well as with HNO_3._ 14 The groups of Weber and Duncan have established a significant amount of data based on the infrared photodissociation spectroscopy of carbon dioxide solvated metal centers M^+/−^(CO_2_)_*n*_ (M=Au, Ag, Co, Ni, Cu, Mg, Fe, Si, V, Al, Bi, TiO),^15^, ^16^, ^17^, ^18^, ^19^, ^20^, ^21^, ^22^, ^23^, ^24^, ^25^, ^26^, ^27^, ^28^, ^29^, ^30^ where CO_2_ is activated by charge transfer to form the carbon dioxide radical anion CO_2_^.−^ or the oxalate dianion C_2_O_4_^2−^. Mackenzie et al. studied the fundamental binding motifs and activation of CO_2_ in cationic metal–CO_2_ complexes M^+^(CO_2_)_*n*_ (M=Co, Rh, Ir), metal oxide clusters MO_2_^+^(CO_2_)_*n*_ (M=Nb, Ta), and in platinum clusters Pt_*n*_^−^ (*n*=4–7) by infrared spectroscopy.^31^, ^32^, ^33^ Bowen and co‐workers showed, by using photoelectron spectroscopy, that anionic complexes of coinage metals and CO_2_ (MCO_2_)^−^ are present as chemisorbed (M=Ag, Au) or physisorbed isomers (M=Cu, Au). Sanov and co‐workers reported evidence for charge transfer to solvent transitions in photoelectron imaging studies of (CO_2_)_*n*_(H_2_O)_*m*_^−^ after excitation at 400 nm. The same group reported photodissociation of CO_2_^.−^ in water clusters.^34^, ^35^

Compared with neutral CO_2_, the symmetric *ν*_s_ and antisymmetric *ν*_as_ C−O stretching vibrations of CO_2_^.−^ are considerably redshifted. The weakening of the C−O bonds, owing to the additional electron in an antibonding molecular orbital, leads to a redshift of both stretching modes.^2^ The excess electron at the carbon atom bends the molecule to a mean angle of 135°, decreasing the difference between *ν*_s_ and *ν*_as_.^3^ The two bands of CO_2_^.−^ in solid neon and argon were observed with vibrational spectroscopy by the groups of Jacox and Andrews, respectively.^36^, ^37^ The Nagata group published vibrational spectroscopy data for small hydrated CO_2_^.−^(H_2_O)_*n*_ clusters in the O−H stretch region above 2800 cm^−1^.^10^, ^38^ They found that CO_2_^.−^(H_2_O) forms a ring structure with two equivalent hydrogen bonds, and two H_2_O molecules are independently bound to the oxygen atoms of the CO_2_^.−^ in CO_2_^.−^(H_2_O)_2_. For [(CO_2_)_*n*_(H_2_O)]^.−^, additional IR absorption bands appear at *n*=4, which are assigned to the bending overtone and the hydrogen‐bonded O−H vibration of H_2_O bound to CO_2_^.−^ through a single O−H⋅⋅⋅O linkage.^38^

Liu et al. performed a detailed theoretical study on localization and time evolution of the excess electron in heterogeneous CO_2_–H_2_O systems.^39^ Their calculations show that hydrogen bonds are not only formed with the oxygen atoms of CO_2_, but also with the carbon atom. Furthermore, they suggest that CO_2_^.−^ is localized inside the cluster with four to seven H_2_O molecules coordinated to the carbon dioxide radical anion. The reactivity of this species is heavily influenced by the surrounding hydrogen‐bond network, which calls for a detailed vibrational spectroscopic analysis of CO_2_^.−^(H_2_O)_*n*_ clusters.

The details of the interaction between ions and water are ideally investigated through microhydration studies, adding water molecules one at a time. Asmis and co‐workers studied how different anions^40^, ^41^ and dianions^42^, ^43^ behave upon stepwise hydration by using infrared spectroscopy combined with quantum chemical calculations. Special interest was devoted to the number of water molecules needed to saturate the first solvation shell. In the HCO_3_^−^(H_2_O)_*n*_ system, a maximum of five water molecules interact directly with the bicarbonate anion,^41^ whereas for the SO_4_^2−^(H_2_O)_*n*_ dianion, twelve water molecules are needed to close the first solvation shell.^42^ These results show that vibrational spectroscopy is a powerful tool to elucidate the structure of the water network surrounding the ionic core. Williams and co‐workers investigated how different core ions influence the hydrogen‐bond network in large water clusters up to *n*≈550, far beyond the first solvation shell.^44^, ^45^, ^46^, ^47^, ^48^, ^49^, ^50^, ^51^ They found that the free O−H band, resulting from the vibration of water molecules with at least one hydrogen atom not involved in hydrogen bonding, redshifts and increases in intensity with increasing positive charge.^44^ Their work demonstrates long‐range solvation effects upon hydration of different ions. In a recent temperature‐controlled experiment with SO_4_^2−^(H_2_O)_*n*_, these ion–water interactions were seen at temperatures that are relevant to Earth's atmosphere.^48^ Thermochemical properties^52^, ^53^, ^54^, ^55^ as well as infrared spectroscopic signatures^56^, ^57^, ^58^ have been frequently investigated as a function of cluster size to determine the transition to bulk‐like behavior of hydrated ions. Along these lines, we investigate size‐selected CO_2_^.−^(H_2_O)_*n*_ clusters, *n*≤61, by using infrared photodissociation spectroscopy in the range 1150–2220 cm^−1^ at *T*=80 K.

## Results and Discussion

**Figure 1:**
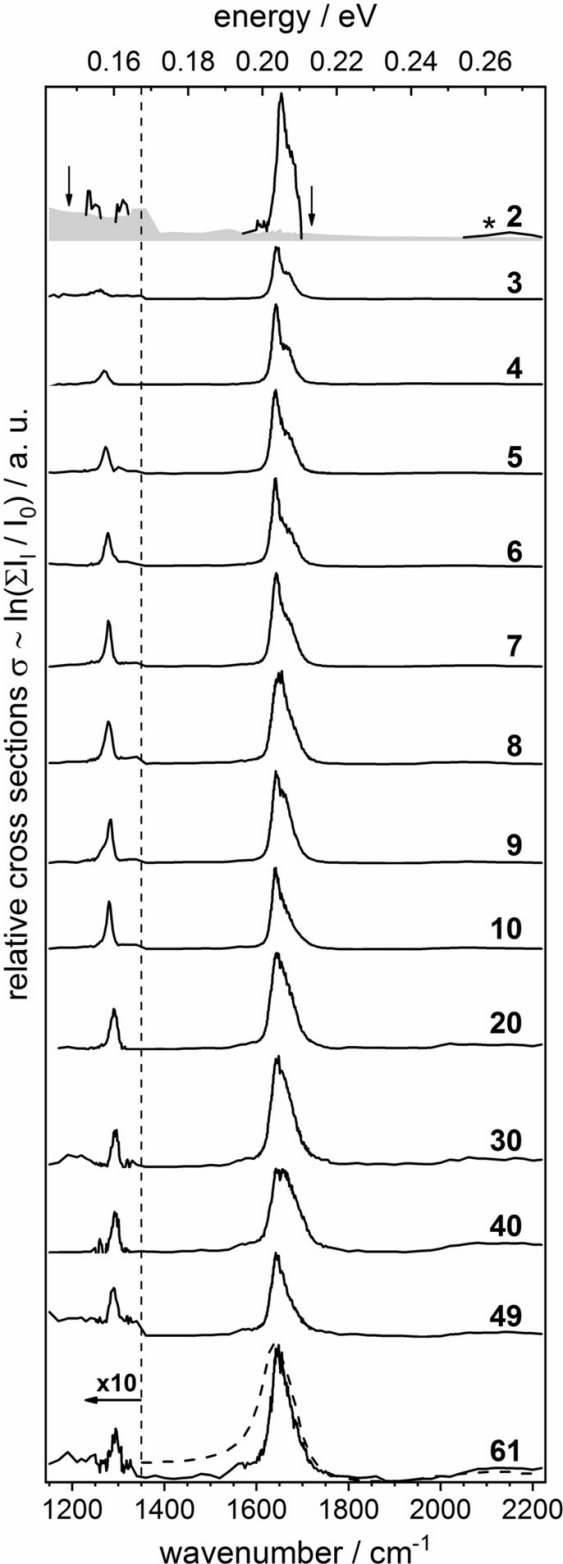
Infrared spectra of CO_2_^.−^(H_2_O)_*n*_ for *n*=2–61, along with the noise level shown in gray. The two absorption lines are the symmetric C−O stretching vibration starting at 1243 cm^−1^ for *n*=2 and the region around 1650 cm^−1^ where the antisymmetric C−O stretching and water bending modes overlap. The asterisk in the *n*=2 panel indicates a band that is only slightly above the noise level and not expected for the small cluster size. It could be caused by signal fluctuations between two reference measurements (see the Experimental Section). The two arrows in the *n*=2 panel show the calculated positions for *ν*_s_ (1193 cm^−1^) and *ν*_as_ (1720 cm^−1^) of gas‐phase CO_2_^.−^. The dashed line shows the IR spectrum of liquid water, scaled to the maximum intensity of the *n*=61 spectrum.^60^

Figure 1 shows the measured IR spectra of CO_2_^.−^(H_2_O)_*n*_, *n*=2–61, at *T*=80 K by using infrared action spectroscopy, assuming single photon absorption for the calculation of relative cross sections. This is correct only for those clusters with a relatively high internal energy, which decay upon absorption of a single photon. The majority of clusters may require two or three IR photons for dissociation, especially at the low‐energy end of the spectrum. However, to account for the frequency‐dependent laser power and the variable irradiation time, the one‐photon cross section is still useful. To derive absolute IR absorption cross sections, master equation modeling is required, which goes beyond the scope of the current work.

**Figure 2:**
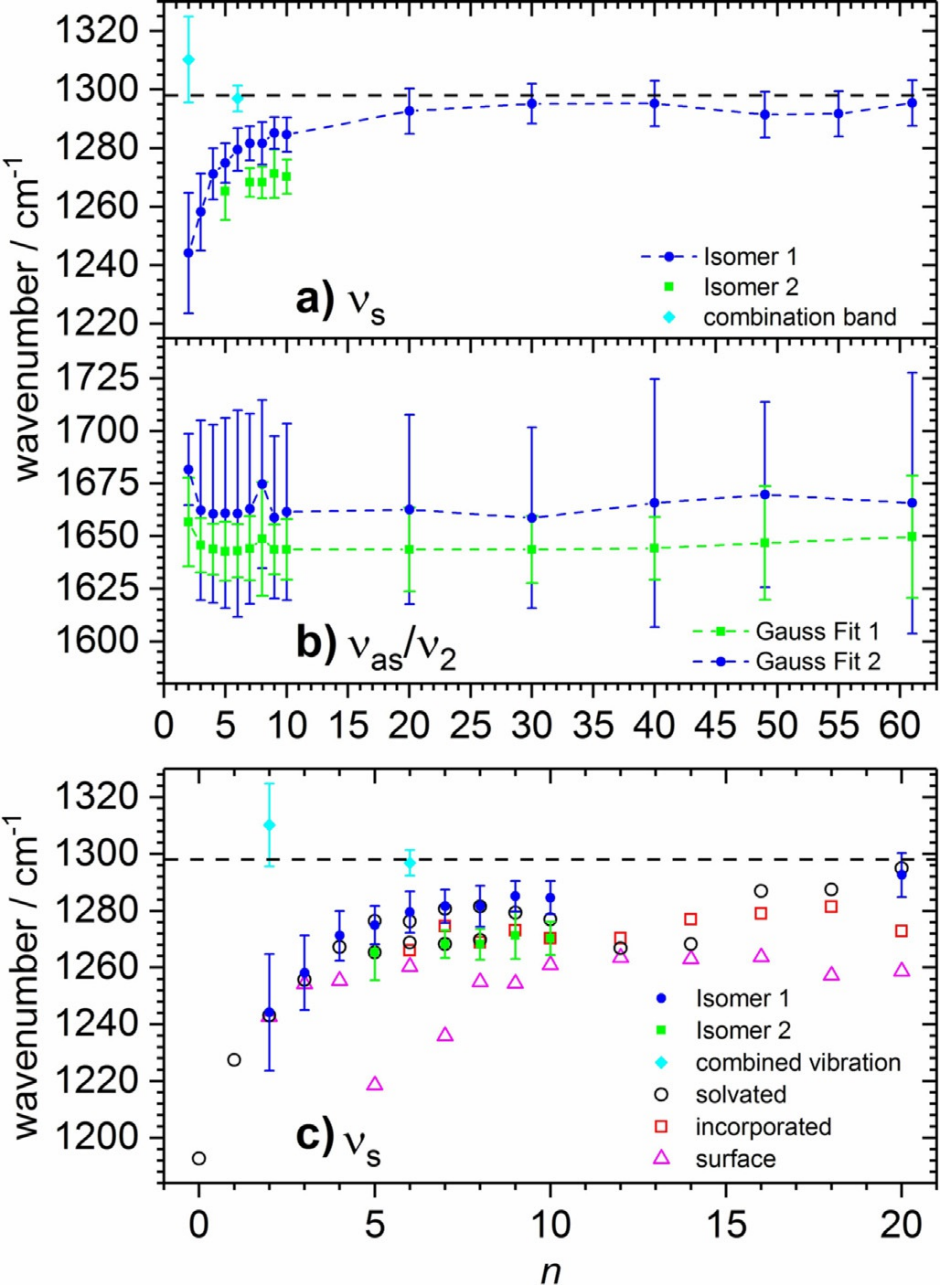
Size dependence of the *ν*_s_ and *ν*_as_/*ν*_2_ absorption bands. a) Position and bandwidth as error bars of the Gaussian fits for *ν*_s_. b) Position and bandwidth of the two most prominent Gaussian fits for *ν*_as_/*ν*_2_ are displayed. The dashed black lines indicate the position of *ν*_s_ in liquid water (1298 cm^−1^).^61^ c) *ν*_s_ vibrational frequencies calculated at the B3LYP/6–311++G** level in the *n*=0–20 region are shown with a scaling factor of 0.977. The diamonds for *n*=2, 6 are interpreted as combinations bands and are discussed in the text.

For a more quantitative analysis, asymmetric peaks are fitted with Gaussian distributions. The most prominent contributing Gaussian distributions are given in Figure 2, with peak positions and line widths as error bars plotted against cluster size. For *n*=2, the cluster decays through electron detachment,^59^ and its spectrum was derived from the signal depletion upon photon absorption; together with the low intensity of CO_2_^.−^(H_2_O)_2_, this leads to a significantly lower signal/noise ratio than for clusters *n*>2, which decay by loss of water molecules.

The low‐energy part of the spectrum at 1150–1350 cm^−1^ shows the symmetric CO_2_^.−^ stretching mode *ν*_s_. The band position is sensitive to the hydrogen‐bond network surrounding the carbon dioxide radical anion, featuring a strong dependence on cluster size, see Figure 2 a and Figure SI‐1 (in the Supporting Information). In the cluster size range of *n*=2–20, the band position shifts to the blue with increasing cluster size, which is attributed to the stabilization of the highest occupied molecular orbital (HOMO) by hydration, leading to a higher force constant (see also Figure SI‐6 in the Supporting Information). For larger clusters (*n*=20–61), the band position is close to the bulk value. For the CO_2_^.−^(H_2_O)_61_ cluster, *ν*_s_ is centered at 1296 cm^−1^, only 2 cm^−1^ away from the value of CO_2_^.−^ in liquid water, which was recently determined as 1298 cm^−1^ by pulse radiolysis time‐resolved resonance Raman spectroscopy.^61^ However, already for *n*=20, *ν*_s_ lies at 1293 cm^−1^, within 5 cm^−1^ of the bulk value. We can conclude that the bulk hydration environment of CO_2_^.−^ is largely developed for CO_2_^.−^(H_2_O)_20_. Although the ion is still located at the cluster surface, the most important interactions with water molecules are present, which are responsible for the frequency shift.

For the particularly stable, magic cluster sizes *n*=49 and *n*=55, a small redshift of approximately 4 cm^−1^ is observed, indicating a slight destabilization of the HOMO in exchange for an increased rigidity of the hydrogen‐bond network. These are, however, small effects, well within the line width. For *n*=2, evidence for a second peak at 1310 cm^−1^ is found, albeit only slightly above noise level. For clusters *n*=5–10, the asymmetry of the peaks indicates contributions of different isomers or from combination bands. Both features are discussed below with the aid of quantum chemical calculations. The irregularities in the 1200–1350 cm^−1^ region for some clusters, in particular *n*=30, 49, and 61, could not be assigned to specific isomers or combination bands and may be noise or a weak water absorption. Accidental spatial alignment of oscillators at a specific cluster size may afford intense combination bands. However, at the low‐frequency end of the spectral range of the laser system, the power drops, and the noise level goes up substantially. Therefore, and owing to the lower intensity of the symmetric C−O stretching mode, irradiation times of 1–2 s were chosen below 1350 cm^−1^, compared with 0.5 s for the major part of the spectrum.

The antisymmetric stretching mode of CO_2_^.−^ is observed around 1650 cm^−1^, strongly coupled to the water bending mode *ν*_2_, denoted *ν*_as_/*ν*_2_. The band is strongly asymmetric, with the higher energy components being more blueshifted with increasing cluster size. The overall band position, however, is only mildly affected by cluster size, with a small shift of 10 cm^−1^ for the measured cluster size range. The *ν*_as_/*ν*_2_ absorption band was fitted with Gaussian functions (see Figure SI‐2 in the Supporting Information); in Figure 2 b, we show the position and full width at half maximum (FWHM) of the two most prominent contributions to the fits. The individual Gaussian functions, however, cannot be assigned to specific vibrational modes owing to the strong coupling of *ν*_as_ and *ν*_2_. In particular, the normal mode analysis of our quantum chemical calculations (see below) does not yield a single mode where only the CO_2_^.−^ atoms are in motion. Instead, several normal modes of the cluster with frequencies around 1650 cm^−1^ have both CO_2_^.−^ and several H_2_O molecules oscillating. The strength of this band scales approximately linearly with cluster size, as expected with the increasing number of water bending modes (Figure SI‐3 in the Supporting Information).

For larger clusters, an additional broad, weak band appears at approximately 2100 cm^−1^, which is assigned to a combination band of H_2_O bending *ν*_2_ (1638 cm^−1^), H_2_O libration *ν*_L2_ (395 cm^−1^), and bending of H_2_O triplets *ν*_T2_ (50 cm^−1^) known from bulk liquid water.^62^, ^63^, ^64^ Its intensity overall increases with cluster size, with a pronounced exception at *n*=49, a cluster size with increased stability, see Figure SI‐4 (in the Supporting Information). In this cluster size region, a strong even–odd oscillation of the ion signal is observed. The integrated peak areas are shown in Figure SI‐5 (in the Supporting Information) as a function of cluster size. Relative to *n*=61, the area reaches 75 % already at *n*=30, and drops to 31 % at *n*=49, lower than the *n*=20 value of 42 %. This largely parallels the behavior of the water bending/C−O stretching peak in Figure SI‐3 (in the Supporting Information), which indicates that, owing to its increased stability, the *n*=49 cluster requires more photons for dissociation than typical. In Figure 1, we also compare the spectrum of the largest cluster (*n*=61) with the IR spectrum of liquid water measured at room temperature.^60^ Both the intense peak at approximately 1650 cm^−1^ and the broad peak at around 2100 cm^−1^ converge to the bulk spectrum, whereas the width of the *ν*_as_/*ν*_2_ band is still smaller than in the bulk.

To get further insight into the hydration of CO_2_^.−^, we calculated various CO_2_^.−^(H_2_O)_*n*_ structures, *n*=0–20, at the B3LYP/6–311++G** level of theory. In the CO_2_^.−^ ion, the calculations predict two features at 1193 and 1720 cm^−1^, assigned to the symmetric *ν*_s_ and antisymmetric *ν*_as_ stretching vibrations, respectively. The third CO_2_^.−^ vibration at 661 cm^−1^ corresponds to bending of the ion. Note that for gas‐phase CO_2_^.−^, an additional structure with a more loosely bound electron was found, reflecting its metastability.^65^ This structure is, however, not relevant here, as CO_2_^.−^ is readily stabilized by water molecules (see above).

**Figure 3:**
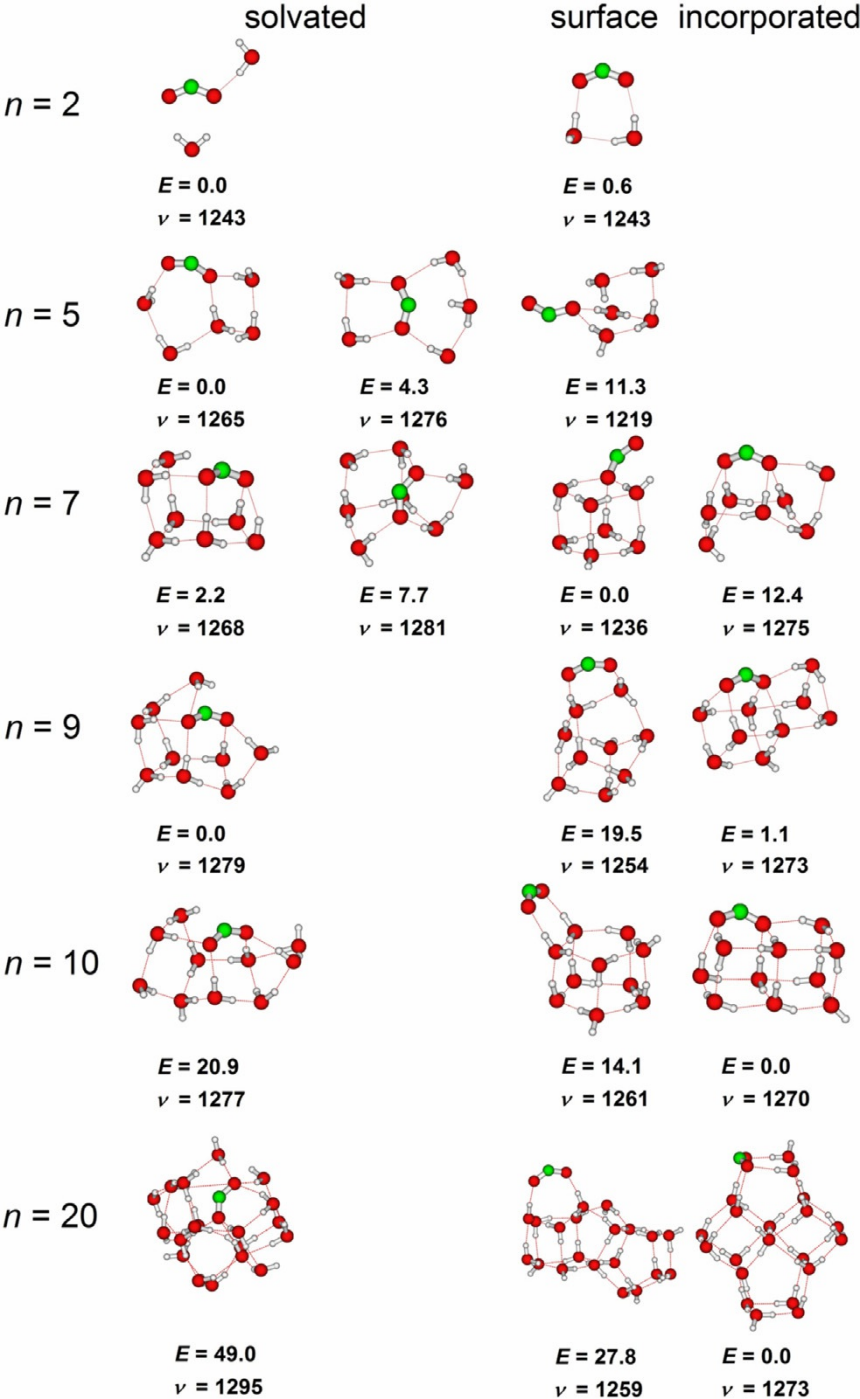
Selected structures optimized at the B3LYP/6–311++G** level, along with their relative energies in kJ mol^−1^ and *ν*_s_ (scaled by 0.977). See the Supporting Information for all optimized structures.

For the hydrated species, three hydration motifs were considered: (i) structures with extensively hydrated CO_2_^.−^ created so as to maximize the number of CO_2_^.−^⋅⋅⋅H_2_O interactions, preferably including the C⋅⋅⋅H interaction (further denoted as “solvated”); (ii) structures with CO_2_^.−^ added on the surface of a compact water cluster (“surface”); (iii) clusters in which CO_2_^.−^ is built into the water cluster structure (“incorporated”), that is, with CO_2_^.−^ assuming at a position that would be otherwise reserved for water molecules in a neutral water cluster, without necessarily the C⋅⋅⋅H interaction present. The latter group can be expected to represent usually the lowest‐energy structure at 0 K. Selected cluster sizes are shown in Figure 3, and all results for *n*=0–20 are available in the Supporting Information (Figure SI‐7).

Within experimental accuracy, the calculated position of the *ν*_s_ vibration matches quantitatively with the measured one in the whole *n*=2–20 range (Figure 2 c). The “solvated” structures with more pronounced C⋅⋅⋅H_2_O interaction seem to follow the experimental values more closely compared with the “incorporated” structures with CO_2_^.−^ positioned on the cluster surface (the difference is, however, within error limits). Within the precision of the computational method, mainly with respect to the accuracy of the DFT approach, it cannot be judged which cluster motif is preferred at a finite temperature. The *ν*_s_ frequency for “surface” structures with CO_2_^.−^ attached to an already formed water cluster, on the other hand, lies outside the error limits of the experimental values. This is more pronounced when the CO_2_^.−^ is incorporated into the water cluster only by one oxygen atom (see clusters with *n*=5, 7 in Figures 2 c and 3). Therefore, we can conclude that the IR spectra document a strong interaction between CO_2_^.−^ and water. Note that CO_2_^.−^ is not fully solvated even for the largest cluster considered computationally (*n*=20) owing to the insufficient size of the cluster. However, CO_2_^.−^ in the “solvated” isomer already interacts with a considerable number of water molecules.

The *n*=2 feature at 1310 cm^−1^, if real, can be assigned to a combination band of the CO_2_^.−^ bending mode at 712 cm^−1^ with the water libration at 650 cm^−1^ for the “solvated” structure type. For *n*=5, 7–10, the minority contribution to the symmetric stretching peak is assigned to a different isomer of the solvated type, and candidates for these isomers are present in the calculations. In line with this argument, the contribution is most pronounced for *n*=9, where the calculated energy difference between the two lowest‐lying isomers is only 1.1 kJ mol^−1^. For *n*=6, the situation is different, the peak exhibits a shoulder on its blue end, and we could not find candidate isomers to explain this feature. As the lowest‐lying structure is a relatively strained cube, we suggest that this particular geometry facilitates coupling of modes, for example, again the CO_2_^.−^ bending mode at around 700–730 cm^−1^ with the water librations below 700 cm^−1^.

To investigate the effect of the C⋅⋅⋅H_2_O interaction on the vibrational frequency, we further optimized 27 different structures for the CO_2_^.−^(H_2_O)_10_ cluster (sampled from a molecular dynamics run at 300 K starting from the lowest‐energy structure in Figure 3). The C⋅⋅⋅H radial distribution function shows a low‐intensity peak at about 2.1 Å, indicating a rather weak interaction, compared with 1.9 Å for the O⋅⋅⋅H radial distribution function (Figure SI‐8 in the Supporting Information). More importantly, the optimized geometries show that the position of the *ν*_s_ frequency (an average of 1273 cm^−1^ with standard deviation of 13 cm^−1^) is independent of the C⋅⋅⋅H distance in the respective local minimum (varying within 2.0–2.8 Å, see Figure SI‐9 in the Supporting Information). A clear distinction of these two binding motifs cannot be obtained based on the position of the *ν*_s_ band.

## Conclusion

We have measured the IR spectra of CO_2_^.−^(H_2_O)_*n*_, *n*=2–61, clusters trapped at a temperature of 80 K. We have shown that the symmetric stretch of the CO_2_^.−^ anion approaches the bulk value already for *n*=20. Analysis of the antisymmetric stretch is hindered by its coupling with the water bending vibration. Its shift, however, seems to be limited within the measured range. The IR spectrum of CO_2_^.−^(H_2_O)_61_ approaches the spectrum of CO_2_^.−^ in bulk aqueous solution. Quantum chemical calculations reproduce quantitatively the position of the symmetric CO_2_^.−^ vibration and suggest that the presence of the C⋅⋅⋅H interaction has a rather limited effect on the IR spectrum in the studied region.

## Experimental and Computational Methods

The experiments were performed with a modified Bruker/Spectrospin CMS47X FT‐ICR (ion cyclotron resonance) mass spectrometer described in detail elsewhere,^55^, ^66^, ^67^ see also the Supporting Information for further details. Hydrated carbon dioxide radical anions CO_2_^.−^(H_2_O)_*n*_ were generated in a laser vaporization source.^68^, ^69^, ^70^ A gas mixture of helium with traces of CO_2_ and water vapor was pulsed into the source region through a piezoelectric valve and expanded into the UHV region (*p*(UHV)<3×10^−10^ mbar) of the instrument. The cell was cooled by liquid nitrogen to *T*≈80 K to minimize blackbody infrared radiative dissociation (BIRD).^71^, ^72^ The beam of an EKSPLA NT273‐XIR optical parametric oscillator laser system was coupled to the cell covering the 4500–12000 nm region (833–2222 cm^−1^, linewidth <1 cm^−1^, 1000 Hz repetition rate, pulse duration <10 ns). The wavelength was calibrated by a HighFinesse Laser Spectrum Analyzer IR‐III. Spectra are recorded by action spectroscopy, reaction (1).

(1) $$\mathrm{CO}{}_{2}{}^{•}{}^{-}\left(H{}_{2}O\right){}_{n}+m\;h\nu {}_{\mathrm{IR}}\to \mathrm{CO}{}_{2}{}^{•}{}^{-}\left(H{}_{2}O\right){}_{n-1}+H{}_{2}O$$

The number of photons that are needed to evaporate a water molecule ranges from one to three, as discussed in the Supporting Information (Figure SI‐10).^73^, ^74^ For the presentation of a realistic spectrum that accounts for laser energy and irradiation time, the single photon cross‐section *σ* is calculated by a modified Lambert–Beer's law:

(2) $$I_{0}=\left(\sum_{i=0}^{l}I_{l}\right)e^{-\frac{\sigma \lambda Pt}{hcA}-k}$$

where *I*_0_ is the intensity of the precursor, *I_l_* is the intensity of the fragments, *λ* is the wavelength, *P* is the laser power, *t* is the irradiation time, *h* is the Planck constant, *A* is the area of the laser beam, and *k* is an empirical factor, which corrects for the contribution of BIRD and cell warming effects caused by the laser.

For optimization and harmonic frequency calculations, the B3LYP/6–311++G** method was used. The scaling factor of 0.977 was chosen as to match the experimental frequency of the symmetric CO_2_^.−^ stretch for *n=*3. A molecular dynamics run was performed for CO_2_^.−^(H_2_O)_10_ at a constant temperature of 300 K on the revPBE potential energy surface, employing the Nosé–Hoover thermostat and the time step of 0.5 fs, with the total running time of 15 ps. In total, 30 geometries were picked in the constant interval of 0.5 ps and optimized into 27 different structures. Room temperature, i.e. 300 K was chosen in the MD run to sample the potential energy surface efficiently. All geometry optimization were performed with the Gaussian suite of programs.^75^ The MD simulation was performed with the Quickstep module of the CP2K suite of programs.^76^ A triple‐zeta Gaussian basis set augmented with diffuse functions plus the Goedecker–Teter–Hutter pseudopotential (with charge density cutoff of 280 Ry) for an auxiliary planewave basis set (TZV2P‐MOLOPT‐GTH) were used. Dispersion interactions were corrected with the Grimme D3 method (with Becke–Johnson damping). The cluster ion was placed at the center of a simulation box with the parameters of 16×16×16 Å^3^, corrected with the Martyna and Tuckerman Poisson solver.

## Conflict of interest

The authors declare no conflict of interest.

## Supporting information

As a service to our authors and readers, this journal provides supporting information supplied by the authors. Such materials are peer reviewed and may be re‐organized for online delivery, but are not copy‐edited or typeset. Technical support issues arising from supporting information (other than missing files) should be addressed to the authors.

Supplementary

## References

[1] M. Aresta , A. Dibenedetto , Dalton Trans. 2007, 2975–2992. 17622414 10.1039/b700658f

[2] L. G. Dodson , M. C. Thompson , J. M. Weber , Annu. Rev. Phys. Chem. 2018, 69, 231–252. 29490208 10.1146/annurev-physchem-050317-021122

[3] J. M. Weber , Int. Rev. Phys. Chem. 2014, 33, 489–519.

[4] M. Knapp , O. Echt , D. Kreisle , T. D. Märk , E. Recknagel , Chem. Phys. Lett. 1986, 126, 225–231.

[5] D. Schröder , C. A. Schalley , J. N. Harvey , H. Schwarz , Int. J. Mass Spectrom. 1999, 185–187, 25–35.

[6] C. D. Cooper , R. N. Compton , Chem. Phys. Lipids 1972, 14, 29.

[7] J. W. Shin , N. I. Hammer , M. A. Johnson , H. Schneider , A. Gloss , J. M. Weber , J. Phys. Chem. A 2005, 109, 3146–3152. 16833642 10.1021/jp050092k

[8] T. Tsukuda , M. A. Johnson , T. Nagata , Chem. Phys. Lett. 1997, 268, 429–433.

[9] C. E. Klots , J. Chem. Phys. 1979, 71, 4172.

[10] A. Muraoka , Y. Inokuchi , N. Nishi , T. Nagata , J. Chem. Phys. 2005, 122, 094303. 15836124 10.1063/1.1850896

[11] R. F. Höckendorf , K. Fischmann , Q. Hao , C. van der Linde , O. P. Balaj , C.-K. Siu , M. K. Beyer , Int. J. Mass Spectrom. 2013, 354–355, 175–180.

[12] A. Akhgarnusch , R. F. Höckendorf , Q. Hao , K. P. Jäger , C.-K. Siu , M. K. Beyer , Angew. Chem. Int. Ed. 2013, 52, 9327–9330; 10.1002/anie.201302827 23843335

[13] A. Herburger , M. Ončák , E. Barwa , C. van der Linde , M. K. Beyer , Int. J. Mass Spectrom. 2019, 435, 101–106. 10.1016/j.ijms.2018.10.019 PMC7116384 33209089

[14] J. Lengyel , C. van der Linde , A. Akhgarnusch , M. K. Beyer , Int. J. Mass Spectrom. 2017, 418, 101–106.

[15] B. J. Knurr , J. M. Weber , J. Phys. Chem. A 2014, 118, 4056–4062. 24835499 10.1021/jp503194v

[16] B. J. Knurr , J. M. Weber , J. Phys. Chem. A 2014, 118, 8753–8757. 25184823 10.1021/jp507149u

[17] B. J. Knurr , J. M. Weber , J. Phys. Chem. A 2014, 118, 10246–10251. 25317936 10.1021/jp508219y

[18] B. J. Knurr , J. M. Weber , J. Phys. Chem. A 2015, 119, 843–850. 25590796 10.1021/jp5108608

[19] G. Gregoire , N. R. Brinkmann , D. van Heijnsbergen , H. F. Schaefer , M. A. Duncan , J. Phys. Chem. A 2003, 107, 218–227.

[20] G. Gregoire , M. A. Duncan , J. Chem. Phys. 2002, 117, 2120.

[21] G. Gregoire , J. Velasquez , M. A. Duncan , Chem. Phys. Lett. 2001, 349, 451–457.

[22] J. B. Jaeger , T. D. Jaeger , N. R. Brinkmann , H. F. Schaefer , M. A. Duncan , Can. J. Chem. 2004, 82, 934–946.

[23] N. R. Walker , G. A. Grieves , R. S. Walters , M. A. Duncan , Chem. Phys. Lett. 2003, 380, 230–236.

[24] N. R. Walker , R. S. Walters , M. A. Duncan , J. Chem. Phys. 2004, 120, 10037. 15268025 10.1063/1.1730217

[25] N. R. Walker , R. S. Walters , G. A. Grieves , M. A. Duncan , J. Chem. Phys. 2004, 121, 10498. 15549932 10.1063/1.1806821

[26] R. S. Walters , N. R. Brinkmann , H. F. Schaefer , M. A. Duncan , J. Phys. Chem. A 2003, 107, 7396–7405.

[27] B. J. Knurr , J. M. Weber , J. Am. Chem. Soc. 2012, 134, 18804–18808. 23098336 10.1021/ja308991a

[28] B. J. Knurr , J. M. Weber , J. Phys. Chem. A 2013, 117, 10764–10771. 24074242 10.1021/jp407646t

[29] M. C. Thompson , J. Ramsay , J. M. Weber , Angew. Chem. Int. Ed. 2016, 55, 15171–15174; 10.1002/anie.201607445 27730755

[30] L. G. Dodson , M. C. Thompson , J. M. Weber , J. Phys. Chem. A 2018, 122, 6909–6917. 30088932 10.1021/acs.jpca.8b06229

[31] A. Iskra , A. S. Gentleman , A. Kartouzian , M. J. Kent , A. P. Sharp , S. R. Mackenzie , J. Phys. Chem. A 2017, 121, 133–140. 27992215 10.1021/acs.jpca.6b10902

[32] A. Iskra , A. S. Gentleman , E. M. Cunningham , S. R. Mackenzie , Int. J. Mass Spectrom. 2019, 435, 93–100.

[33] A. E. Green , J. Justen , W. Schöllkopf , A. S. Gentleman , A. Fielicke , S. R. Mackenzie , Angew. Chem. Int. Ed. 2018, 57, 14822–14826; 10.1002/anie.201809099 30207020

[34] T. Habteyes , L. Velarde , A. Sanov , Chem. Phys. Lett. 2006, 424, 268–272.

[35] T. Habteyes , L. Velarde , A. Sanov , J. Chem. Phys. 2007, 126, 154301. 17461620 10.1063/1.2717932

[36] W. E. Thompson , M. E. Jacox , J. Chem. Phys. 1999, 111, 4487–4496.

[37] M. F. Zhou , L. Andrews , J. Chem. Phys. 1999, 110, 2414–2422.

[38] A. Muraoka , Y. Inokuchi , N. I. Hammer , J. W. Shin , M. A. Johnson , T. Nagata , J. Phys. Chem. A 2009, 113, 8942–8948. 19603758 10.1021/jp903578e

[39] P. Liu , J. Zhao , J. Liu , M. Zhang , Y. Bu , J. Chem. Phys. 2014, 140, 044318. 25669534 10.1063/1.4863343

[40] D. J. Goebbert , E. Garand , T. Wende , R. Bergmann , G. Meijer , K. R. Asmis , D. M. Neumark , J. Phys. Chem. A 2009, 113, 7584–7592. 19445493 10.1021/jp9017103

[41] E. Garand , T. Wende , D. J. Goebbert , R. Bergmann , G. Meijer , D. M. Neumark , K. R. Asmis , J. Am. Chem. Soc. 2010, 132, 849–856. 20030394 10.1021/ja9093132

[42] J. Zhou , G. Santambrogio , M. Brümmer , D. T. Moore , L. Wöste , G. Meijer , D. M. Neumark , K. R. Asmis , J. Chem. Phys. 2006, 125, 111102. 16999457 10.1063/1.2351675

[43] Y. Miller , G. M. Chaban , J. Zhou , K. R. Asmis , D. M. Neumark , R. B. Gerber , J. Chem. Phys. 2007, 127, 094305. 17824737 10.1063/1.2764074

[44] J. T. O′Brien , E. R. Williams , J. Am. Chem. Soc. 2012, 134, 10228–10236. 22616651 10.1021/ja303191r

[45] J. T. O′Brien , J. S. Prell , M. F. Bush , E. R. Williams , J. Am. Chem. Soc. 2010, 132, 8248–8249. 20518523 10.1021/ja1024113

[46] J. S. Prell , J. T. O′Brien , E. R. Williams , J. Am. Chem. Soc. 2011, 133, 4810–4818. 21405134 10.1021/ja108341t

[47] M. F. Bush , J. T. O'Brien , J. S. Prell , C.-C. Wu , R. J. Saykally , E. R. Williams , J. Am. Chem. Soc. 2009, 131, 13270–13277. 19754186 10.1021/ja901011x

[48] M. J. DiTucci , C. N. Stachl , E. R. Williams , Chem. Sci. 2018, 9, 3970–3977. 29780530 10.1039/c8sc00854j PMC5942037

[49] R. J. Cooper , M. J. DiTucci , T. M. Chang , E. R. Williams , J. Am. Chem. Soc. 2016, 138, 96–99. 26714195 10.1021/jacs.5b11880

[50] M. J. DiTucci , S. Heiles , E. R. Williams , J. Am. Chem. Soc. 2015, 137, 1650–1657. 25569605 10.1021/ja5119545

[51] R. J. Cooper , J. T. O'Brien , T. M. Chang , E. R. Williams , Chem. Sci. 2017, 8, 5201–5213. 28970907 10.1039/c7sc00481h PMC5618692

[52] W. A. Donald , R. D. Leib , M. Demireva , B. Negru , D. M. Neumark , E. R. Williams , J. Phys. Chem. A 2011, 115, 2–12. 21142113 10.1021/jp107547r

[53] W. A. Donald , R. D. Leib , J. T. O′Brien , E. R. Williams , Chem. Eur. J. 2009, 15, 5926–5934. 19440999 10.1002/chem.200900334 PMC2757329

[54] A. Akhgarnusch , R. F. Höckendorf , M. K. Beyer , J. Phys. Chem. A 2015, 119, 9978–9985. 26356833 10.1021/acs.jpca.5b06975

[55] A. Akhgarnusch , W. K. Tang , H. Zhang , C.-K. Siu , M. K. Beyer , Phys. Chem. Chem. Phys. 2016, 18, 23528–23537. 27498686 10.1039/c6cp03324e

[56] J. M. Weber , J. A. Kelley , S. B. Nielsen , P. Ayotte , M. A. Johnson , Science 2000, 287, 2461–2463. 10741960 10.1126/science.287.5462.2461

[57] W. H. Robertson , M. A. Johnson , Annu. Rev. Phys. Chem. 2003, 54, 173–213. 12626732 10.1146/annurev.physchem.54.011002.103801

[58] C. Chaudhuri , Y.-S. Wang , J. C. Jiang , Y. T. Lee , H.-C. Chang , G. Niedner-Schatteburg , Mol. Phys. 2001, 99, 1161–1173.

[59] O. P. Balaj , C.-K. Siu , I. Balteanu , M. K. Beyer , V. E. Bondybey , Chem. Eur. J. 2004, 10, 4822–4830. 15372683 10.1002/chem.200400416

[60] Coblentz Society, Inc., “Evaluated Infrared Reference Spectra” in NIST Chemistry WebBook, NIST Standard Reference Database Number 69, (Eds.: P. J. Linstrom, W. G. Mallard), National Institute of Standards and Technology, Gaithersburg, MD, (retrieved February 12, 2019).

[61] I. Janik , G. N. R. Tripathi , J. Chem. Phys. 2016, 144, 154307. 27389220 10.1063/1.4946868

[62] J.-J. Max , C. Chapados , J. Chem. Phys. 2002, 116, 4626–4642.

[63] H. R. Zelsmann , J. Mol. Struct. 1995, 350, 95–114.

[64] K. H. Tsai , T.-M. Wu , Chem. Phys. Lett. 2006, 417, 389–394.

[65] V. K. Voora , A. Kairalapova , T. Sommerfeld , K. D. Jordan , J. Chem. Phys. 2017, 147, 214114. 29221378 10.1063/1.4991497

[66] R. F. Höckendorf , O. P. Balaj , C. van der Linde , M. K. Beyer , Phys. Chem. Chem. Phys. 2010, 12, 3772–3779. 20358037 10.1039/b921395c

[67] C. Berg , T. Schindler , G. Niedner-Schatteburg , V. E. Bondybey , J. Chem. Phys. 1995, 102, 4870–4884.

[68] V. E. Bondybey , J. H. English , J. Chem. Phys. 1981, 74, 6978–6979.

[69] T. G. Dietz , M. A. Duncan , D. E. Powers , R. E. Smalley , J. Chem. Phys. 1981, 74, 6511–6512.

[70] S. Maruyama , L. R. Anderson , R. E. Smalley , Rev. Sci. Instrum. 1990, 61, 3686–3693.

[71] R. C. Dunbar , Mass Spectrom. Rev. 2004, 23, 127–158. 14732935 10.1002/mas.10074

[72] O. P. Balaj , C. B. Berg , S. J. Reitmeier , V. E. Bondybey , M. K. Beyer , Int. J. Mass Spectrom. 2009, 279, 5–9.

[73] N. K. Bersenkowitsch , M. Ončák , J. Heller , C. van der Linde , M. K. Beyer , Chem. Eur. J. 2018, 24, 12433–12443. 29979470 10.1002/chem.201803017 PMC6120481

[74] J. Heller , M. Ončák , N. K. Bersenkowitsch , C. van der Linde , M. K. Beyer , Eur. J. Mass Spectrom. 2019, 25, 122–132. 10.1177/1469066718803307 PMC7100558 30284923

[75] Gaussian 16, Revision A.03, M. J. Frisch, G. W. Trucks, H. B. Schlegel, G. E. Scuseria, M. A. Robb, J. R. Cheeseman, G. Scalmani, V. Barone, B. Mennucci, G. A. Petersson, H. Nakatsuji, M. Caricato, X. Li, H. P. Hratchian, A. F. Izmaylov, J. Bloino, G. Zheng, J. L. Sonnenberg, M. Hada, M. Ehara, K. Toyota, R. Fukuda, J. Hasegawa, M. Ishida, T. Nakajima, Y. Honda, O. Kitao, H. Nakai, T. Vreven, J. A. Montgomery, Jr., J. E. Peralta, F. Ogliaro, M. Bearpark, J. J. Heyd, E. Brothers, K. N. Kudin, V. N. Staroverov, R. Kobayashi, J. Normand, K. Raghavachari, A. Rendell, J. C. Burant, S. S. Iyengar, J. Tomasi, M. Cossi, N. Rega, J. M. Millam, M. Klene, J. E. Knox, J. B. Cross, V. Bakken, C. Adamo, J. Jaramillo, R. Gomperts, R. E. Stratmann, O. Yazyev, A. J. Austin, R. Cammi, C. Pomelli, J. W. Ochterski, R. L. Martin, K. Morokuma, V. G. Zakrzewski, G. A. Voth, P. Salvador, J. J. Dannenberg, S. Dapprich, A. D. Daniels, Ö. Farkas, J. B. Foresman, J. V. Ortiz, J. Cioslowski, D. J. Fox, Gaussian, Inc. Wallingford CT, 2016.

[76] J. VandeVondele , M. Krack , F. Mohamed , M. Parrinello , T. Chassaing , J. Hutter , Comput. Phys. Commun. 2005, 167, 103–128.

