# Supplementary material for: Infrared Spectroscopy of Size‐Selected Hydrated Carbon Dioxide Radical Anions CO2 .−(H2O)n (n=2–61) in the C−O Stretch Region
Source: Chemistry. 2019 Jul 1;25(43):10165–71. doi: 10.1002/chem.201901650 (PMC6771497; doi:10.1002/chem.201901650)
Supplement: Supplementary file 1 — Supplementary [file CHEM-25-10165-s001.pdf]

CHEMISTRY
A European Journal

Supporting Information

Infrared Spectroscopy of Size-Selected Hydrated Carbon Dioxide Radical Anions $CO_2^{\bullet-}(H_2O)_n$ ($n=2$–61) in the C–O Stretch Region

Andreas Herburger,[a] Milan Ončák,[a] Chi-Kit Siu,[b] Ephrem G. Demissie,[b] Jakob Heller,[a] Wai Kit Tang,[b] and Martin K. Beyer*[a]

chem_201901650_sm_miscellaneous_information.pdf

Supporting Information

Contents

S-1 Data Fitting 2

S-2 Magic numbers 5

S-3 Experimental Setup 6

S-4 Further Theoretical Data 8

S-5 Multiphoton analysis 11

S-6 Cartesian coordinates of optimized ions and molecules 12

S-7 References 46

S-1 Data Fitting

S-1A Symmetric C–O stretch region

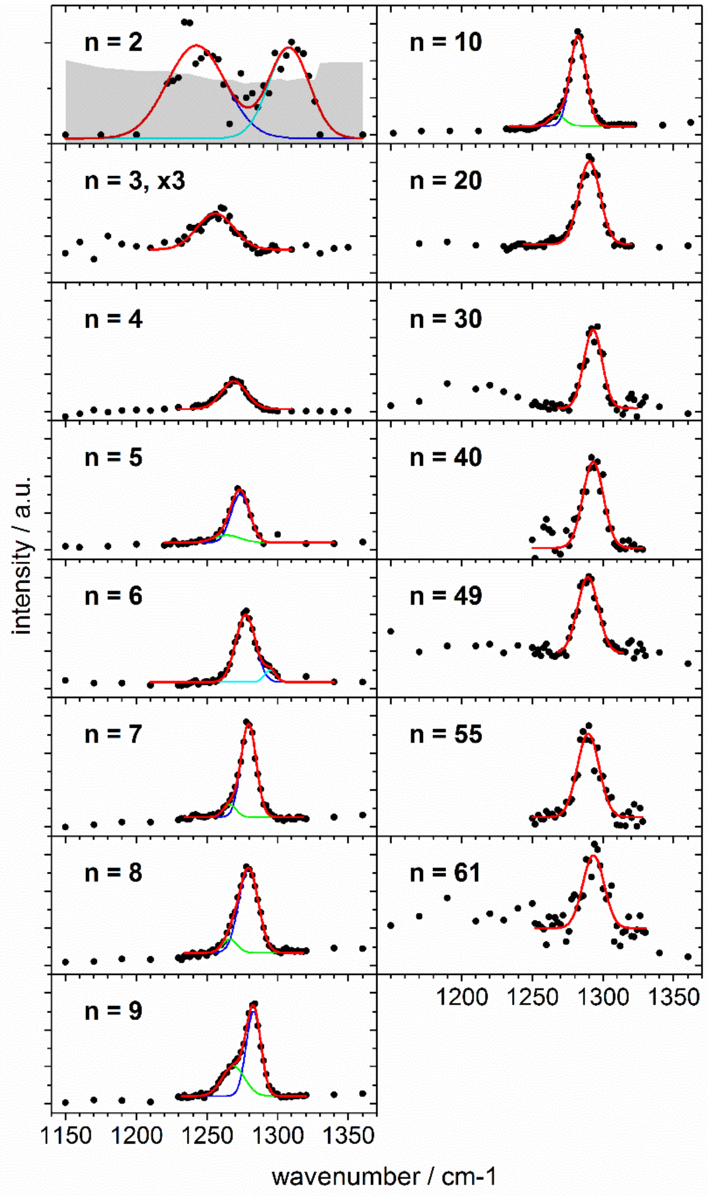

Figure SI-1. Gaussian fits for the symmetric C–O stretch vibration. For n = 5–10, two Gaussians are necessary to fit the data. The asymmetry is explained by presence of several isomers (see Figure 2). The hydrogen bond network around the $CO_2^{\bullet-}$ does not change significantly for clusters with $n \geq 20$ and the position of the absorption stays constant within the error limits of the experiment.

S-1B Antisymmetric C–O stretch/water bend region

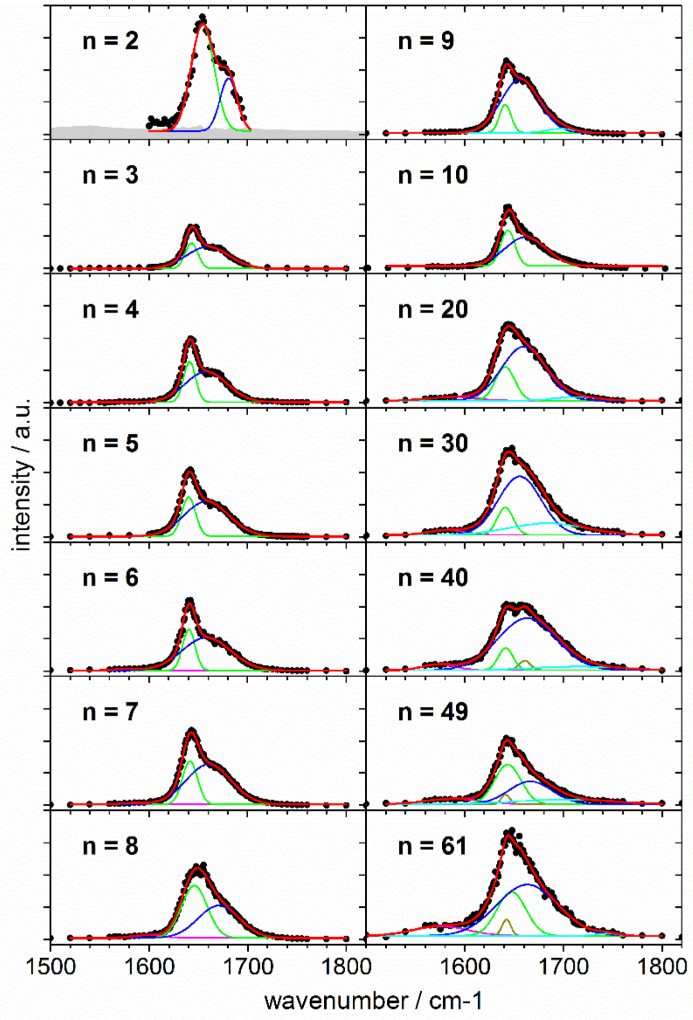

Figure SI-2. Gaussian fits for the antisymmetric C–O stretch/H_2O bend region. With increasing cluster size, more Gaussians are needed to fit the data, and the contribution of water absorptions to the total absorption grows. Different structural isomers may also be present, shifting the C–O stretch vibration.

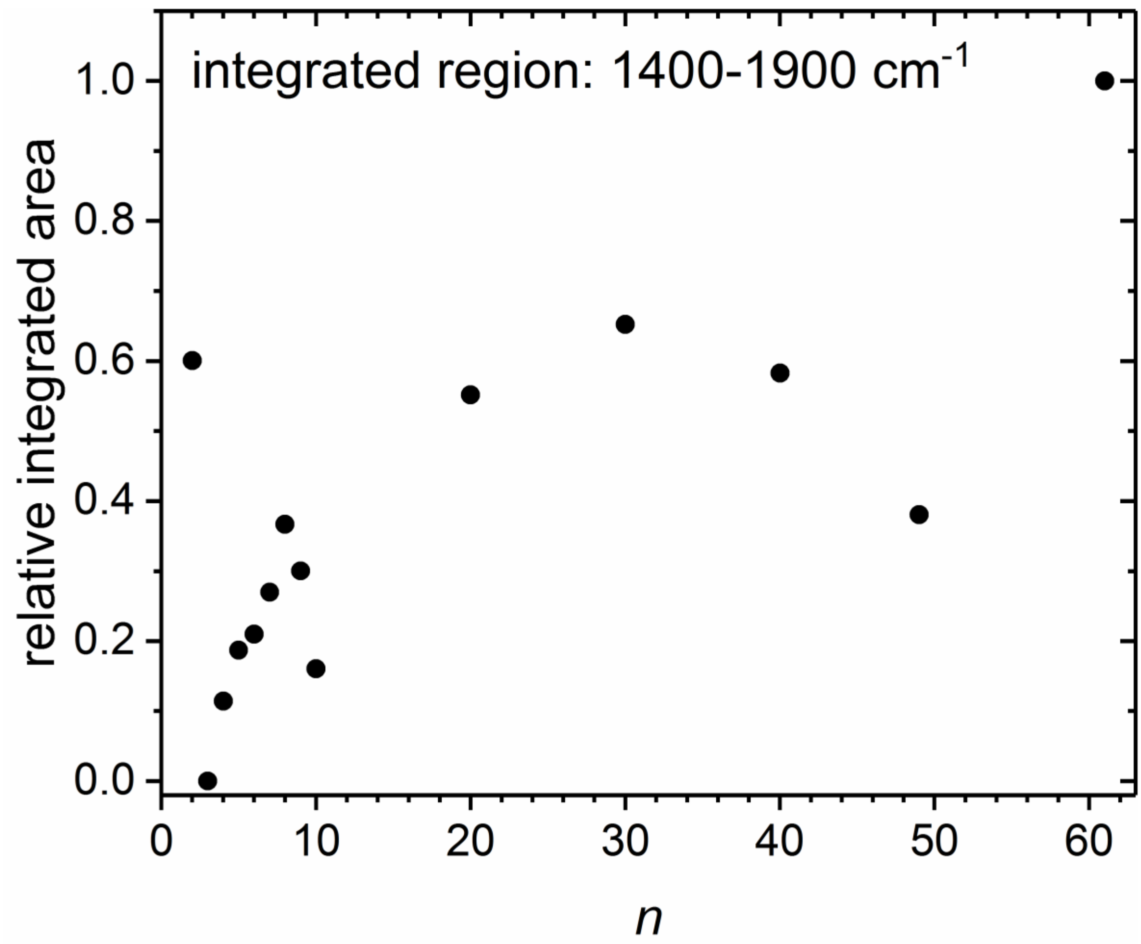

Figure SI-3. Integrated region of the antisymmetric C–O stretch/water bend vibrations. The strength scales approximately linear with cluster size, with a pronounced deviation for the magic n = 49 cluster.

S-2 Magic numbers

In Figure 2, a small redshift for the symmetric C-O vibration is observed between n = 40 and 61. Figure SI-4 shows the cluster distribution of $CO_2^{\bullet -}(H_2O)_n$ tuned to maximize the intensity of $CO_2^{\bullet -}(H_2O)_{49}$. Starting at n = 49, every second cluster size exhibits a significantly higher ion signal than the neighboring clusters. At around n = 59, this trend stops. The higher stability, hence a different structure, causes the redshift.

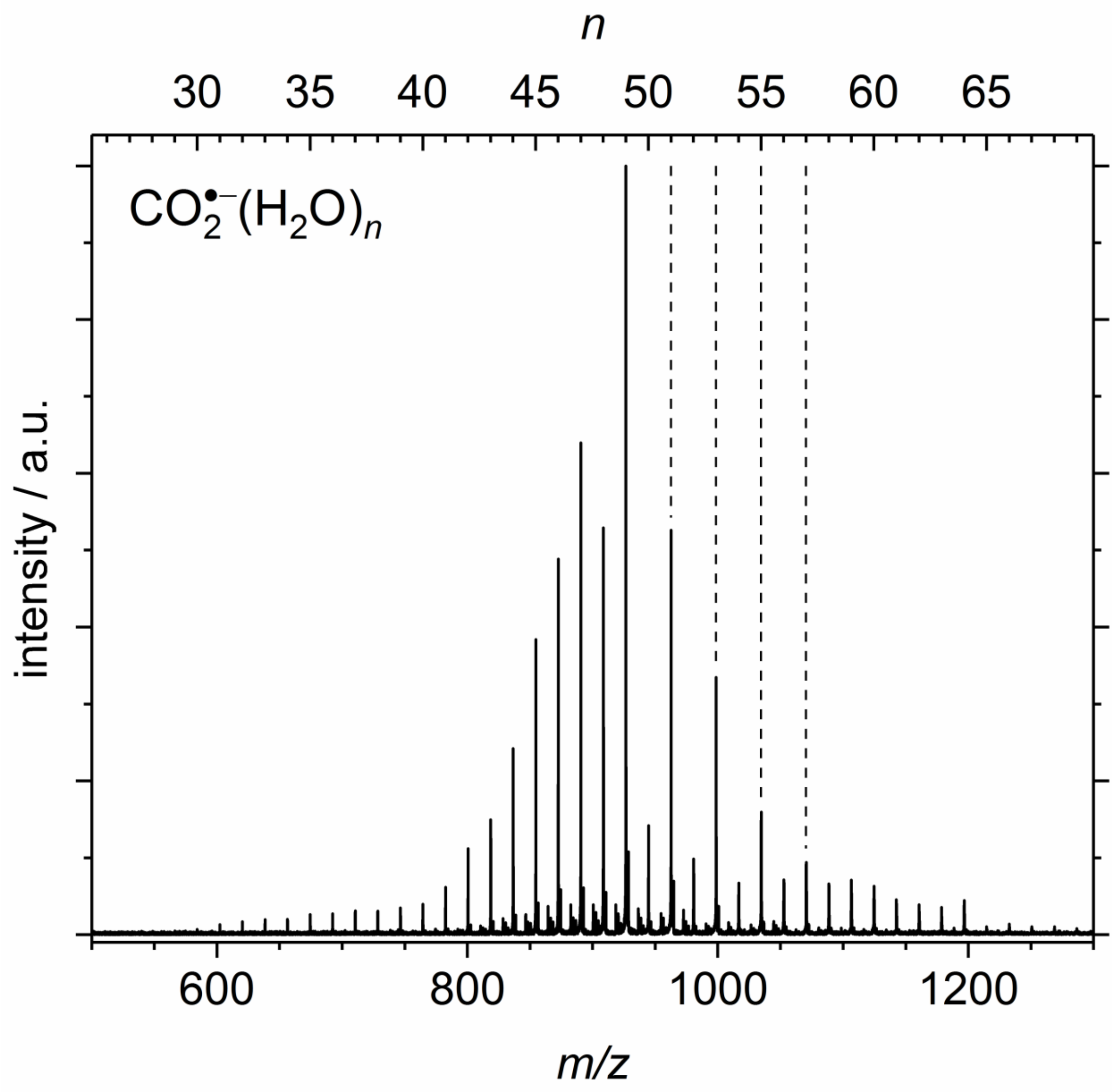

Figure SI-4. Mass spectrum of $CO_2^{\bullet -}(H_2O)_n$ centered at n = 49. The clusters with 49, 51, 53, 55 and 57 water molecules show a significantly higher intensity than their neighbors, indicating a higher stability. A similar trend has been observed in anionic water clusters $(H_2O)_n^-$.[1] The small peaks between the $CO_2^{\bullet -}(H_2O)_n$ clusters are hydrated electrons $(H_2O)_n^-$, which do not interfere with the experiment.

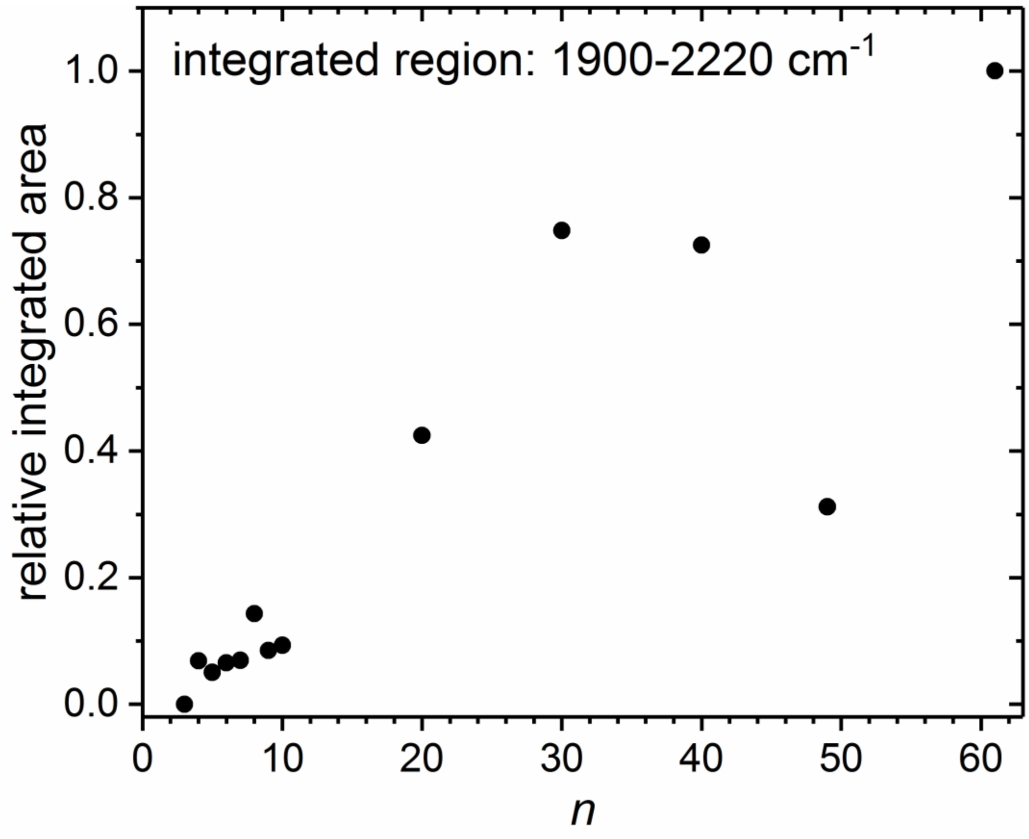

Figure SI-5. Integrated area in the region of the combination band above 1900 cm^{-1}. The area increases with cluster size with the exception of n = 49, which can be explained by the high stability of this cluster (Figure SI-4).

S-3 Experimental Setup

The experiments are performed on a modified Bruker/Spectrospin CMS47X FT-ICR mass spectrometer described in detail elsewhere.[2–4] Hydrated carbon dioxide radical anions $CO_2^{\bullet-}(H_2O)_n$ are generated in a laser vaporization source.[5–7] Electrons are formed by focusing the beam of a Litron Nano S 60-30 on a rotating zinc target, operating at 30 Hz. A gas mixture of helium with traces of CO_2 and water vapor is pulsed into the source region via a piezoelectric valve. $CO_2^{\bullet-}(H_2O)_n$ ions are guided into the ICR cell via a system of electrostatic lenses where they are mass selected. The cell is cooled to T ~ 80 K to minimize blackbody infrared radiative dissociation (BIRD) while the ions are stored. The beam of an EKSPLA NT273-XIR optical parametric oscillator laser system is coupled into the cell covering the region of 4500–12000 nm (833–2222 cm^{-1}, linewidth < 1 cm^{-1}, 1000 Hz repetition rate, pulse duration < 10 ns). The

wavelength is calibrated by a HighFinesse Laser Spectrum Analyzer IR-III. Vibrational infrared multi photon dissociation (IRMPD) spectra are recorded by action spectroscopy, reaction 1.

$CO_2^{\bullet-}(H_2O)_n + m \cdot h\nu_{IR} \rightarrow CO_2^{\bullet-}(H_2O)_{n-1} + H_2O$ (1)

The single photon cross section σ is calculated by a modified Lambert Beer's law:

$$I_0 = \left(\sum_{i=0}^{l} I_l\right) e^{-\frac{\sigma\lambda P t}{hcA} - k} \qquad (2)$$

I_0 is the intensity of the precursor, I_l the intensity of the fragments, λ the wavelength, P the laser power, t the irradiation time, h the Planck constant, A the area of the laser beam and k an empirical factor which corrects for the contribution of BIRD and cell warming effects caused by the laser. The laser power is measured after each data point. Ions are irradiated for 0.5 s in the 1350–2220 cm^{-1} region and for 1–2 s in the 1150–1349 cm^{-1} region due to the weaker symmetric C–O absorption compared to the antisymmetric C–O stretching and H_2O bending modes.

Photodissociation of $CO_2^{\bullet-}(H_2O)_2$ proceeds by electron detachment. Since electrons are not directly detected in the experiment, the detachement intensity is measured indirectly by recording a reference spectrum without laser irradiation every five data points, yielding the total amount of ions in the cell I_{ref}. Ions are well confined by the high magnetic field (4.7 T) and collisions with background gas can be neglected due to the ultra-high vacuum in the ICR cell ($2 \cdot 10^{-10}$ mbar). Excluding other ion loss channels, the contribution of electron detachment I_{detach} is calculated as:

$I_{\mathrm{detach}} = I_{\mathrm{ref}} - I_0$ (3)

S-4 Further Theoretical Data

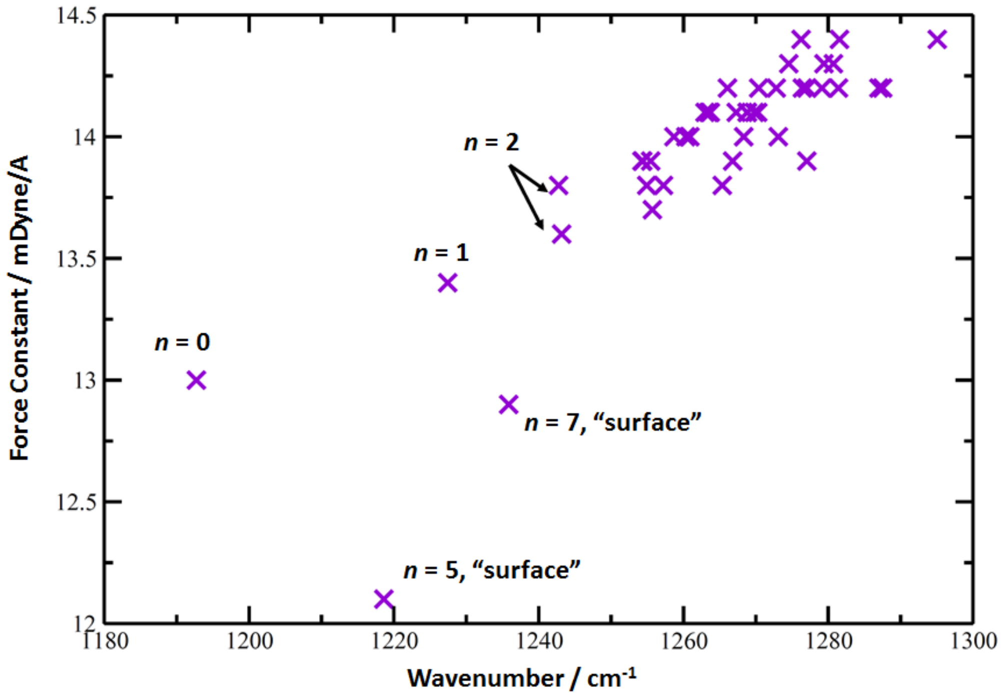

Figure SI-6. Correlation between the ν_s vibration position and the respective force constant for all structures included in Figure SI-7. The isomers with 0–2 water molecules and surface isomers coordinated only by one O atom of CO_2^- (*n* = 5,7) are labeled explicitly. Harmonic frequencies were scaled by a factor of 0.977.

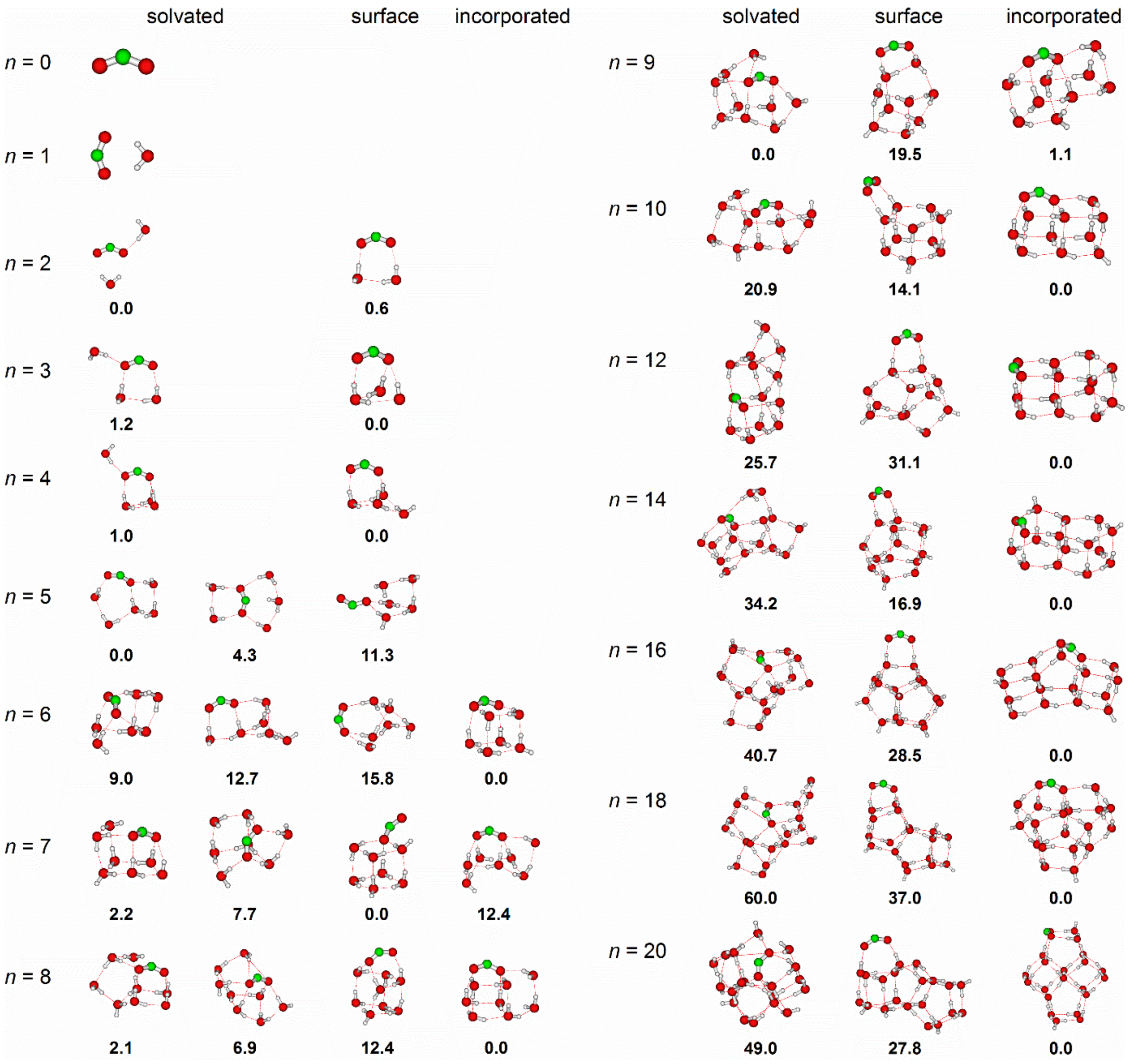

Figure SI-7. All calculated structures optimized at the B3LYP/6-311++G** level, along with their relative energy in kJ/mol.

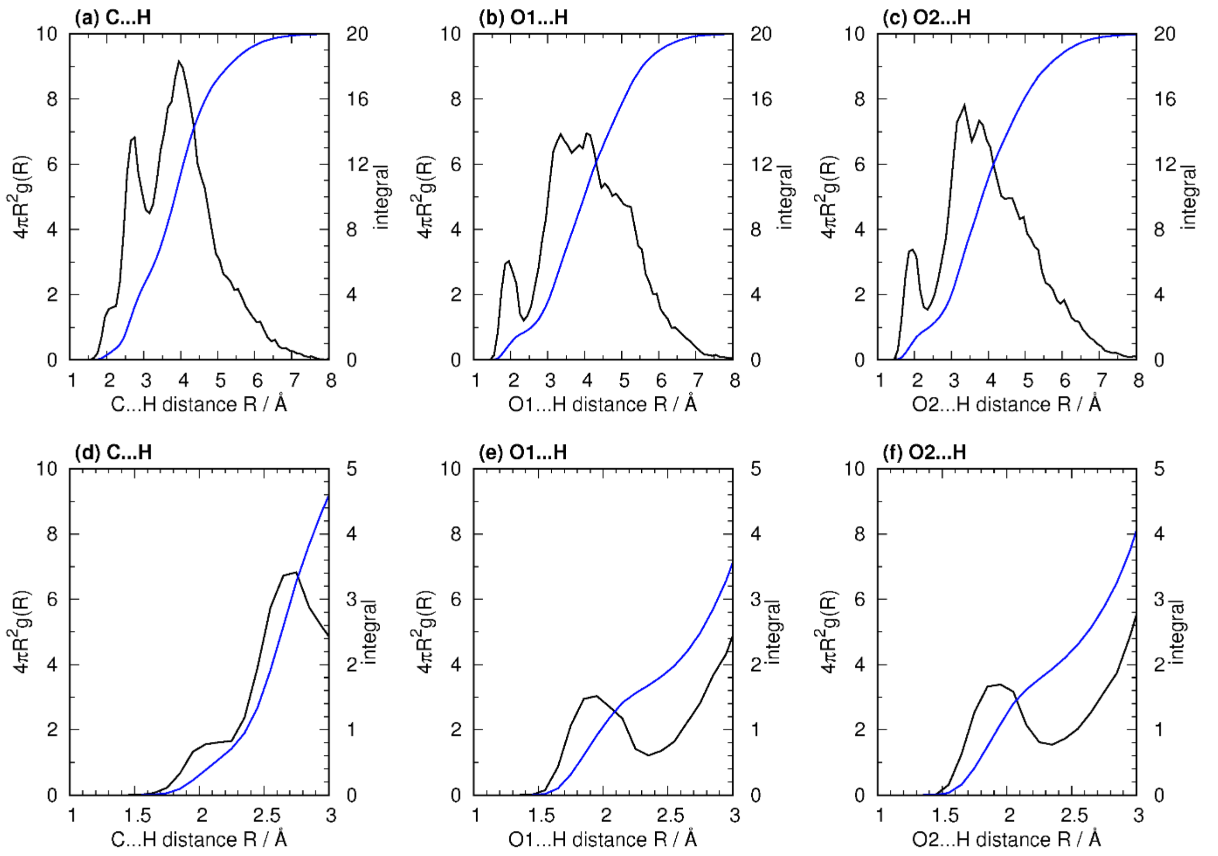

Figure SI-8. Radial distribution functions (RDFs) of a) C…H pair, b) O1…H pair and c) O2…H pair of O1-C-O2 (same RDFs with R within 1–3 Å are shown in d), e) and f), respectively). Calculated from a molecular dynamics run at 300 K on the PBE potential energy surface.

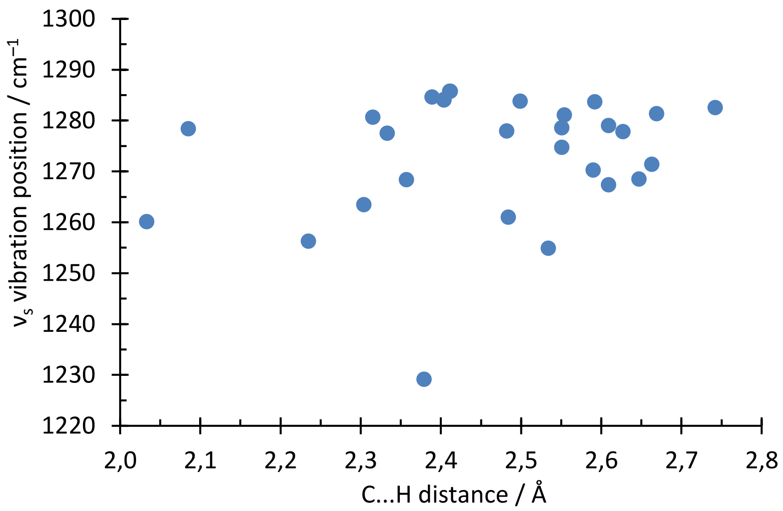

Figure SI-9. Dependence of the ν_s vibration position with respect to the C…H distance in local minima for 27 different structures randomly taken from a molecular dynamics run and optimized at the B3LYP/6-311++G** level of theory. Harmonic frequencies were scaled by a factor of 0.977.

S-5 Multiphoton analysis

Figure SI-10 shows the multiphoton analysis of $CO_2^{\bullet-}(H_2O)_4$. The calculated binding energy of the fourth water molecule is 0.42 eV. In the C–O antisymmetric stretch/H_2O bend region the energy of one photon is approximately 0.2 eV, in the C–O symmetric stretch region 0.16 eV. Depending on the internal energy of the cluster, one to three photons are needed to evaporate a water molecule from the cluster.

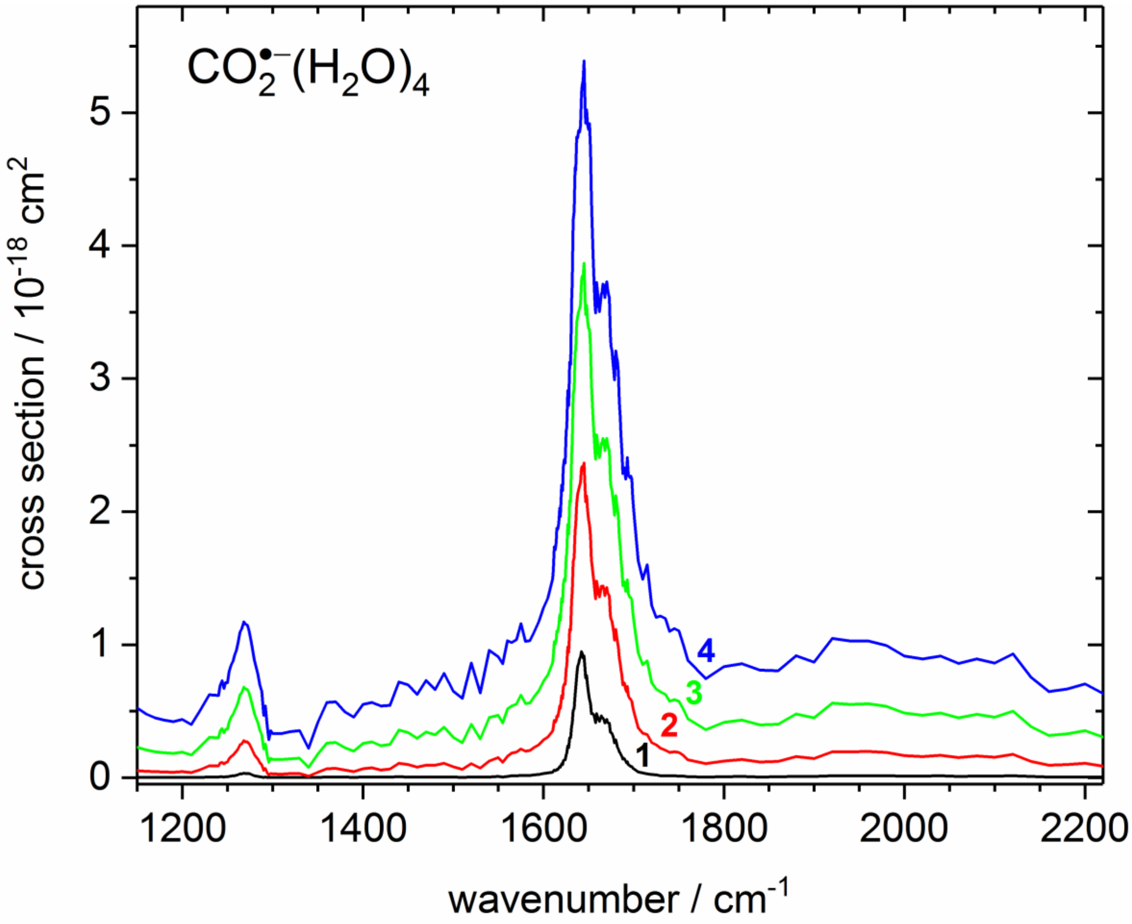

Figure SI-10. Multiphoton analysis of the $n = 4$ cluster. The numbers next to the curves indicate the number of sequentially absorbed photons used for the calculation of the cross section.[8,9]

S-6 Cartesian coordinates of optimized ions and molecules

Cartesian coordinates (in Å) and energies (in Hartree) of ions included in Figures 2 and SI-7, optimized at the B3LYP/6-311++G level of theory.**

CO2-, solv
E = -188.624877
C 0.000000 -0.000000 0.322120
O -0.000000 1.148440 -0.120795
O -0.000000 -1.148440 -0.120795

CO2-.H2O, solv
E = -265.084136
C -1.183400 0.000641 -0.000070
O -0.719287 -1.143901 -0.000035
O -0.717034 1.144313 0.000044
H 1.352146 0.738342 0.000042
O 1.985680 -0.000619 0.000037
H 1.353386 -0.740535 0.000013

CO2-.(H2O)2, solv
E = -341.539568
C -0.044757 0.905121 0.000087
O -1.141529 1.456695 -0.000163
O 0.343725 -0.278139 -0.000121
O -2.471062 -1.178755 -0.000243
H -2.467964 -0.207169 0.000499
H -1.513111 -1.340537 -0.000733
H 2.139594 -0.524072 0.000187
O 3.122626 -0.478451 0.000388
H 3.279932 0.470250 0.000639

CO2-.(H2O)2, surf
E = -341.539352
C -1.699375 0.021360 0.006964
O -1.209739 -1.111666 -0.125550
O -1.256463 1.163523 0.120639
H 0.686279 1.550241 -0.002704
O 1.658848 1.511725 -0.073673
H 1.818762 0.557599 -0.151673
O 1.504834 -1.468028 -0.007275
H 1.584617 -1.636926 0.936261
H 0.526746 -1.363507 -0.136799

CO2-.(H2O)3, solv
E = -417.992748
C 0.197418 -1.464444 0.087344
O 0.817483 -0.376634 0.058283
O -0.985723 -1.776000 0.072110
H -2.483278 -0.440743 -0.115812
O -3.022284 0.365884 -0.199687
H -2.348589 1.064459 -0.193319
O -0.556075 2.008176 0.081323
H -0.492952 2.223873 1.016470
H -0.052416 1.160140 0.005223
O 3.521835 0.263203 -0.086124
H 2.619054 -0.104301 0.033311
H 3.371782 1.006208 -0.677173

CO2-.(H2O)3, surf
E = -417.993202
C -1.858067 -0.516371 0.048257
O -0.904145 -1.313257 0.082814
O -1.961234 0.706470 -0.065081
O 1.453110 -0.251046 1.569173
O 1.543775 -0.763896 -1.361490
O 0.617595 1.886889 -0.244873
H -0.327221 1.627888 -0.178718
H 0.969009 1.258620 -0.895796
H 1.967091 -0.752953 -0.489111
H 0.641822 -1.054088 -1.124231
H 1.263318 0.635635 1.212171
H 0.641566 -0.738161 1.341801

CO2-.(H2O)4, solv
E = -494.445559
C -0.927379 -0.989804 0.064229
O -1.318811 0.183375 -0.045680
O 0.182761 -1.529104 0.090530
O 0.953988 1.924991 -0.224659
O 2.461541 -0.415423 -1.377405
O 2.298296 0.051473 1.542963
H 0.086457 1.475744 -0.161038
H 1.424183 1.403309 -0.894546
H 2.858539 -0.313633 -0.497753
H 1.657884 -0.921672 -1.162353
H 1.890782 0.866119 1.194677
H 1.614363 -0.613331 1.359092
H -4.271512 -0.626908 0.100031
O -4.143852 0.318455 -0.020631
H -3.167803 0.399064 -0.044415

CO2-.(H2O)4, surf
E = -494.445954
C 2.087015 -1.022789 0.042363
O 2.604716 0.086016 -0.082170
O 0.910485 -1.433435 0.099905

O 0.485683 2.030198 -0.124211
O -0.830088 0.227629 1.797239
O -0.844208 0.019442 -1.640472
H 1.322627 1.521135 -0.137984
H 0.025018 1.669748 0.655119
H -1.681945 0.071213 1.355560
H -0.232137 -0.420424 1.378764
H -0.468366 0.871513 -1.336298
H -0.240271 -0.618780 -1.208168
O -3.042014 -0.361291 -0.065645
H -2.323820 -0.197542 -0.727915
H -3.199791 -1.308600 -0.110422

CO2-.(H2O)5, solv
E = -570.895992
C -0.256318 -2.138728 -0.115317
O -1.457912 -2.251834 -0.327270
O 0.457300 -1.284893 0.449576
O 2.846199 1.696012 -0.418223
O 0.260937 1.534564 1.044260
O -3.289355 -0.140842 -0.046600
H -2.662068 -0.877722 -0.214740
H -3.284103 -0.051696 0.911307
H 1.151528 1.743607 0.718137
H 0.191858 0.568949 0.920709
H 3.034745 0.737872 -0.321156
H 2.364452 1.765186 -1.248302
O 3.076009 -1.063842 -0.061985
H 3.261670 -1.678498 -0.777170
H 2.127387 -1.237163 0.178186
H -1.217997 2.286965 0.015736
O -2.009984 2.500252 -0.509680
H -2.495123 1.659535 -0.531422

CO2-.(H2O)5, solv
E = -570.894346
C 0.020889 -0.105992 -0.660501
O -0.343631 1.066625 -0.500853
O -0.481155 -1.218042 -0.480180
O -3.188377 -1.429346 0.179185
O 2.141934 -2.499874 0.775416
O 3.132749 0.048968 -0.464805
O -3.173453 1.525315 0.375516
H -2.249185 1.540324 0.077325
H -3.360156 0.574403 0.432367
H -3.542873 -1.679396 -0.679031
H -2.216255 -1.404282 0.033403
H 1.256662 -2.300378 0.438370
H 2.657609 -1.716208 0.527035
H 2.283119 -0.108747 -0.913868
H 2.973713 0.910763 -0.042363
H 1.038480 2.128093 0.037018
O 1.840126 2.518422 0.449826
H 1.608014 2.594824 1.379916

CO2-.(H2O)5, surf
E = -570.891696
O 1.500579 -0.026837 -0.071489
O -0.060818 2.504850 -0.944850
O -0.206376 -0.156654 1.934846
O -2.312175 1.307963 0.575844
O -2.954644 -1.036279 -1.046799
O -0.499615 -2.022762 -0.272697
C 2.745636 -0.121424 0.130091
H 0.259745 -1.489617 -0.579366
H -0.535291 -1.773744 0.664360
H 0.191865 -0.002066 2.795581
H 0.523015 -0.002962 1.266631
H -2.060388 -1.420686 -0.892206
H -3.568955 -1.707772 -0.738415
H -1.687773 0.855623 1.169081
H -2.653782 0.601526 0.000999
H -0.873615 2.221870 -0.492647
H 0.550124 1.764857 -0.811073
O 3.705704 -0.360092 -0.570290

CO2-.(H2O)6, incorp
E = -647.356190
H 1.576439 -0.397326 -1.528749
H 2.921574 -1.207508 -1.581916
H -0.471482 -0.006639 -1.967997
H 0.308016 1.283965 -1.676843
H -0.643768 2.042727 0.114208
H 0.882569 2.094878 0.318111
H 0.909565 -2.082933 -0.104809
H -0.622833 -2.027438 -0.162851
H 1.422618 0.507970 1.807362
H 2.352457 0.353714 0.594113
H -0.803541 0.039521 1.980893
H 0.002266 -1.249703 1.847768
O 2.235837 -0.910844 -0.977830
O 0.342841 0.469428 -2.228986
O 0.167886 2.433245 -0.259266
O 0.114411 -2.426873 0.336097
O 2.096625 0.989818 1.281316
O -0.064464 -0.441534 2.394224
O -1.913339 -0.800048 -0.989014
O -2.094362 0.793827 0.635343
C -2.486225 -0.034562 -0.195726

CO2-.(H2O)6, solv
E = -647.352781
C 0.116376 -1.179204 -1.354733
O 0.810586 -1.743253 -0.493751
O 0.147081 -0.079298 -1.912413
O 3.126821 -0.647736 0.600545

O 1.883034 1.750660 -0.458663
O -0.349291 1.911109 1.326467
O -0.592129 -0.813006 1.976920
O -2.835644 -1.179656 0.307858
O -2.204894 1.407588 -0.822044
H 0.473182 1.987945 0.802346
H -1.055142 1.918645 0.650655
H -1.508568 1.042946 -1.393026
H -2.621829 0.608568 -0.447665
H 2.483351 1.083174 -0.081645
H 1.362009 1.237269 -1.104932
H 2.386101 -1.115158 0.152064
H 2.918260 -0.723108 1.537089
H -0.464896 0.155762 1.860180
H -0.027577 -1.212585 1.295681
H -2.350425 -1.664026 -0.373741
H -2.177228 -1.095469 1.032045

CO2-.(H2O)6, solv
E = -647.351360
O -1.264661 -2.047510 0.242937
O -1.778614 0.384909 1.766406
O -2.007025 -0.080866 -1.660123
O -4.025719 0.847744 -0.065724
O 2.839309 2.180104 -0.097657
O 0.189654 1.074261 -0.219784
H -0.293020 -2.076833 0.183418
H -1.442657 -1.387888 0.939993
H -1.784948 -0.899769 -1.167065
H -1.256754 0.500827 -1.438664
H 0.927648 1.709694 -0.242617
H 0.658260 0.206396 -0.169376
H -4.684622 0.148026 -0.047560
H -3.362970 0.552039 -0.738673
H -2.603981 0.645884 1.322874
H -1.077968 0.802191 1.230792
H 3.160469 1.246055 -0.148191
H 3.078223 2.459445 0.791096
O 1.489160 -1.349381 -0.019917
C 2.730697 -1.357330 0.025469
O 3.595163 -0.479522 -0.029744

CO2-.(H2O)6, surf
E = -647.350156
O -0.008695 1.388719 1.771263
O 0.105305 2.064096 -1.041581
O -1.440987 -0.060162 -2.089919
O -0.046543 -2.002462 -0.572706
O -1.343278 -1.193177 1.535118
O -3.332809 0.225926 0.188160
H -2.740169 -0.331382 0.734078
H -3.155517 1.122481 0.490190
H -1.401611 -1.895581 2.188214
H -0.808905 -1.563751 0.758595
H -0.417563 0.508982 1.831691
H 0.899203 1.209897 1.458158
H -2.217734 0.015105 -1.511021
H -0.897581 0.721613 -1.853970
H 0.908520 -1.760742 -0.512069
H -0.439474 -1.413369 -1.250916
H 1.011028 1.722004 -0.920129
H -0.206408 2.055923 -0.118842
O 2.609336 -1.286801 -0.255057
C 2.979219 -0.153747 0.057806
O 2.406532 0.930274 0.259620

CO2-.(H2O)7, incorp
E = -723.802827
H 1.416526 -1.940761 0.913912
H 2.744514 -1.662317 0.202721
H 0.812237 0.014790 1.665520
H -0.456442 -0.720757 2.077594
H -1.981642 0.747607 1.322735
H -2.657082 -0.612687 1.079827
H 3.269464 0.124786 -1.228310
H 2.998693 0.851747 0.067061
H 1.149860 -1.261928 -1.263433
H 0.949843 0.240503 -1.090134
H -2.773889 -0.892229 -1.119868
H -2.743400 -2.343528 -0.591711
H -1.670760 1.030828 -1.352915
H -0.903804 -0.070172 -2.087234
O 1.979872 -2.259081 0.177006
O 0.518735 -0.810004 2.086779
O -2.208043 -0.085879 1.768083
O 3.632374 0.237079 -0.340494
O 0.916830 -0.460026 -1.772253
O -3.228244 -1.517595 -0.503299
O -1.805000 0.296220 -1.980052
O 1.111459 1.499377 0.303669
O -1.053123 2.187439 0.155887
C 0.154499 2.298982 0.373603

CO2-.(H2O)7, solv
E = -723.806701
C -0.359205 -0.123986 -1.934786
O 0.156356 -1.221163 -1.663498
O -1.490038 0.352960 -1.836975
O -0.825148 -2.183830 0.834244
O 1.229790 -0.996775 2.042316
O -3.028414 -0.490305 0.504283
O -1.822257 2.069050 0.645591
O 0.841341 1.862390 1.573246
O 2.189739 1.705248 -0.948617
H 0.973398 0.937224 1.842594
H 1.358713 1.944690 0.746906

H 2.589454 0.821513 -0.807408
H 1.441867 1.505775 -1.534568
H 1.418882 -1.430785 2.878616
H 0.422241 -1.452135 1.668416
H -1.658692 -1.665149 0.878002
H -0.534502 -2.055421 -0.091485
H -2.797191 -0.411152 -0.438052
H -2.746066 0.385115 0.837185
H -0.926327 2.060359 1.044571
H -1.667545 1.835113 -0.282334
O 2.738632 -1.023772 -0.471175
H 1.951121 -1.232387 -1.010410
H 2.409866 -1.089267 0.441361

CO2-.(H2O)7, solv
E = -723.804597
C -0.492843 -0.430049 -1.753494
O -0.191463 -1.530672 -1.270896
O -1.469728 0.319894 -1.643066
O 2.326193 -2.249378 -0.374776
O 2.766820 0.265893 0.998661
O 0.206799 0.625027 2.208055
O -0.571005 2.436721 0.225585
O -3.462525 -0.041351 0.309665
O -1.329269 -1.679423 1.395688
O 1.890927 2.056418 -1.085578
H -0.275498 -0.208364 2.042905
H 1.125991 0.455714 1.915351
H 2.737407 -0.595948 0.544718
H 2.628248 0.920627 0.287580
H -2.184930 -1.252634 1.211223
H -0.949923 -1.792938 0.504545
H -2.876054 0.034488 -0.473695
H -3.400733 0.823010 0.729687
H -0.365551 1.849245 0.988986
H -0.996654 1.853166 -0.422568
H 1.483310 -2.009503 -0.818108
H 2.074244 -2.928233 0.258457
H 1.537590 1.432969 -1.734073
H 1.085633 2.373660 -0.620746

CO2-.(H2O)7, surf
E = -723.807547
O 1.069670 -1.435510 1.978672
O -0.899405 -1.884008 -0.078337
O 0.141040 1.303037 2.024451
O 2.107916 2.036621 0.153584
O 2.984857 -0.776158 -0.120451
O 0.205978 1.448713 -1.883645
O 1.114139 -1.160552 -1.979233
H 0.783413 -0.238112 -2.102472
H 0.397592 -1.595367 -1.481085
H -0.595882 1.337308 -1.340901
H 0.869938 1.809930 -1.260569
H -1.261122 -0.952150 -0.041978
H -1.689696 -2.433806 -0.116085
H 0.374978 -1.771731 1.384306
H 1.832098 -1.293759 1.392037
H 3.821461 -1.181173 -0.363953
H 2.351198 -0.962764 -0.870617
H 2.590988 1.195831 0.114282
H 1.471160 1.907089 0.889736
H 0.419251 0.381863 2.190181
H -0.640283 1.214214 1.449383
O -3.912639 -0.291471 -0.063115
C -3.095727 0.608172 -0.020353
O -1.830399 0.626027 0.003056

CO2-.(H2O)8, incorp
E = -800.256522
H 0.758952 -1.221887 1.275743
H 1.805386 -2.070946 0.520333
H 0.398022 0.924924 1.066579
H -0.477034 0.422127 2.208834
H -2.397311 0.919591 1.183092
H -2.382192 -0.571803 1.563887
H 2.760135 -1.118483 -1.300955
H 3.396515 -0.341964 -0.150466
H 2.305844 1.789297 0.075094
H 2.545285 1.193097 1.449478
H 0.588277 -1.287643 -1.004798
H 0.630103 0.150488 -1.527964
H -2.272661 -1.779840 -0.322599
H -1.220503 -2.391669 0.625147
H -2.289554 0.180300 -1.538212
H -1.302757 -0.820195 -2.133212
O 0.857145 -2.039712 0.738169
O 0.459525 0.445982 1.916020
O -2.329037 0.327985 1.949560
O 3.354258 -1.234916 -0.543926
O 3.006493 1.396127 0.623907
O 0.655110 -0.776684 -1.835480
O -2.158571 -2.153061 0.576402
O -2.238323 -0.731568 -1.874860
O 0.417287 1.807149 -0.600211
O -1.854229 1.945062 -0.639550
C -0.700628 2.355612 -0.745041

CO2-.(H2O)8, solv
E = -800.255734
C 0.888790 -1.406388 -1.367704
O 2.095488 -1.239679 -1.189430
O -0.031283 -0.714256 -1.836289
O 0.317699 2.005406 -1.143742
O -1.137335 2.039814 1.050914
O 2.994568 1.461119 -0.604368

O 2.675611 -0.253852 1.627129
O 0.003729 -0.237013 2.535845
O -2.625762 -1.404538 -0.866622
O -0.970029 -2.496570 1.113607
H -0.435607 0.503648 2.085946
H -0.388771 -1.042395 2.138659
H -1.699868 -2.185032 0.535428
H -0.243317 -2.617214 0.483276
H -1.016647 2.880405 1.501933
H -0.540814 2.064664 0.241620
H 1.282884 1.983451 -0.953329
H 0.146414 1.116719 -1.523440
H 2.907738 0.629044 -1.103349
H 2.979741 1.125947 0.314881
H 1.781731 -0.227042 2.031745
H 2.578587 -0.875520 0.890072
H -1.805910 -1.172118 -1.345302
H -3.023633 -0.551622 -0.618472
O -3.524858 1.270132 -0.168079
H -2.748088 1.568963 0.350357
H -3.489793 1.791933 -0.975512

CO2-.(H2O)8, solv
E = -800.253911
C -0.022082 -1.623148 0.738503
O -1.021902 -1.891274 0.062046
O 0.213594 -0.894948 1.705208
O -3.635679 -1.027070 0.616051
O -0.430787 1.894499 1.088058
O -2.878112 1.621581 -0.295983
O -1.279006 0.184140 -2.194132
O 0.913747 1.743968 -1.481085
O 3.354737 0.496223 -0.685684
O 2.380275 0.939633 1.987053
O 2.401509 -2.239864 -1.002955
H 0.556120 1.909295 -0.588270
H 1.798176 1.354265 -1.328413
H 3.199266 0.656497 0.264465
H 3.175722 -0.452947 -0.806641
H -1.329022 1.980982 0.702935
H -0.401284 0.971715 1.393049
H -3.296111 0.844259 0.114797
H -2.431764 1.239829 -1.079108
H -0.446935 0.696136 -2.084518
H -1.167394 -0.583007 -1.612993
H 1.773387 0.176972 2.020793
H 1.778246 1.688542 1.884115
H -2.715027 -1.359810 0.538231
H -4.119083 -1.507064 -0.062899
H 1.681930 -2.205291 -0.334039
H 1.929234 -2.286594 -1.841144

CO2-.(H2O)8, surf
E = -800.251789
O -0.398246 -1.684651 1.702144
O 0.226694 -2.082157 -1.027563
O 3.009798 -1.202330 -0.796767
O 2.037853 1.142184 -1.667472
O -0.771161 0.556020 -1.805784
O -1.025091 2.209970 0.405690
O 1.776718 1.955206 1.075854
O 2.076704 -0.543313 1.904519
H -1.191613 -1.115617 1.664854
H -0.288877 -1.985646 0.777091
H 2.268976 2.568101 1.628366
H 1.908842 1.043674 1.467549
H 2.746076 -0.328881 -1.191870
H 3.805586 -1.486158 -1.253983
H -0.160967 2.152736 0.842754
H -1.597760 1.522286 0.810332
H 2.501034 -0.949187 1.131037
H 1.170211 -0.944022 1.940170
H 1.190088 -1.974904 -1.037547
H -0.120374 -1.216802 -1.320462
H -1.690994 0.213432 -1.826295
H -0.833484 1.217070 -1.077762
H 1.125226 0.966141 -1.963012
H 1.941782 1.610895 -0.817245
O -2.640907 0.030621 1.121174
C -3.394481 -0.339585 0.196593
O -3.343220 -0.288501 -1.030985

CO2-.(H2O)9, incorp
E = -876.708971
H 0.171758 -1.875010 -0.572896
H -0.071582 -3.253379 0.132107
H -0.385621 0.177249 -1.126229
H 0.692083 -0.415739 -2.045501
H 2.337481 0.972326 -1.597693
H 2.850162 -0.469709 -1.449593
H -2.180501 -1.071841 -1.771204
H -3.044384 -1.300388 -0.511104
H -2.214818 -0.811195 1.858686
H -3.320475 0.001775 1.169903
H -2.469885 1.782824 -0.066922
H -3.441913 0.851444 -0.803632
H 0.044524 -1.152655 1.501142
H -0.334451 0.340910 1.379391
H 3.178023 -0.892447 0.744604
H 2.453253 -2.089654 0.116152
H 2.426690 1.214513 1.248102
H 1.757605 0.233608 2.212591
O 0.474015 -2.462310 0.168871
O -0.197859 -0.616424 -1.670074
O 2.424240 0.139065 -2.090022
O -3.099305 -1.099067 -1.463533

O -3.087127 -0.907133 1.441519
O -3.401005 1.496795 -0.078063
O -0.209958 -0.375832 2.032287
O 3.256457 -1.553189 0.025470
O 2.630848 0.500162 1.877871
O -0.530148 1.650835 0.003349
O 1.628424 2.343809 -0.195450
C 0.407232 2.470613 -0.139282

CO2-.(H2O)9, solv
E = -876.709375
C 0.244868 -1.452535 -1.188060
O 0.069215 -0.499663 -1.946865
O 1.160709 -1.844704 -0.451629
O 3.870628 -1.188903 -0.261189
O 1.223430 2.071648 -1.183826
O -1.101069 2.832349 -0.160913
O 2.970334 1.330174 0.832176
O 1.034879 -0.247737 2.139944
O -1.454150 1.021364 2.097262
O -3.371875 -0.563821 0.738919
O -2.541234 0.791179 -1.619524
O -1.936335 -3.056796 0.233625
H -1.358531 1.724480 1.432394
H -2.162297 0.444730 1.739546
H -3.226188 -0.136473 -0.129818
H -3.018601 -1.463263 0.626213
H -1.185739 3.789055 -0.190419
H -0.192407 2.617714 -0.521045
H 1.896907 1.871593 -0.491649
H 0.987272 1.206603 -1.566020
H 3.477535 0.585577 0.462980
H 2.326909 0.889275 1.427608
H 0.150644 0.180972 2.189136
H 0.973976 -0.862761 1.393597
H -1.751277 0.294675 -1.896508
H -2.173858 1.590592 -1.203209
H 2.937028 -1.420641 -0.457345
H 4.134885 -1.823294 0.411690
H -1.320104 -2.657578 -0.417202
H -1.361627 -3.276789 0.974583

CO2-.(H2O)9, surf
E = -876.701932
O 2.090889 1.123789 -1.808822
O 1.038186 -1.331328 -2.010661
O 2.803771 -2.196548 0.133565
O 3.703808 0.334028 0.374901
O 1.651926 1.023500 2.203707
O 0.488386 -1.282288 1.665191
O -1.195192 -1.005027 -0.468927
O -1.880250 1.581664 -0.903524
O 0.547580 2.749529 0.127611
H 3.158499 0.682744 1.106067
H 3.319152 0.737059 -0.427085
H 0.852871 2.283058 0.923588
H 1.014726 2.296753 -0.594279
H -0.213110 -1.184580 0.971913
H 1.184072 -1.810721 1.242913
H 1.606415 -1.837955 -1.407841
H 0.147124 -1.339700 -1.587866
H 1.643714 0.241553 -1.947585
H 2.218748 1.499847 -2.684258
H 1.149899 0.166898 2.059900
H 1.589277 1.222331 3.141998
H 3.251107 -1.315826 0.239018
H 3.478658 -2.871253 0.244709
H -1.360400 -0.046895 -0.666693
H -2.114278 -1.346450 -0.308654
H -2.724136 1.467597 -0.410519
H -1.284509 2.147535 -0.384348
O -3.813566 -1.501098 0.096824
C -4.448721 -0.476155 0.381629
O -4.213726 0.736896 0.365042

CO2-.(H2O)10, incorp
E = -953.167488
H 0.175438 -1.456830 0.775155
H 1.204693 -2.324745 0.013435
H 3.874982 -2.415737 -0.235245
H 3.104627 -1.162409 -0.791473
H -0.094033 0.674774 1.250973
H -0.981495 -0.183527 2.170032
H -2.943784 0.465220 1.438916
H -2.874007 -1.073572 1.412414
H 1.848509 0.025112 2.293345
H 2.998407 -0.609679 1.495159
H 1.963969 0.010406 -2.174083
H 2.880834 0.895231 -1.308058
H 1.637883 2.325648 -0.019823
H 2.716984 1.588094 0.805081
H 0.068342 -0.912644 -1.374144
H -0.076191 0.617718 -1.415664
H -2.854960 -1.740401 -0.726664
H -1.758117 -2.550188 -0.010325
H -2.964131 0.472771 -1.368674
H -1.956593 -0.275062 -2.238044
O 0.256742 -2.109129 0.042143
O 3.115696 -1.851406 -0.065710
O -0.045027 -0.067343 1.887468
O -2.792755 -0.303055 2.013814
O 2.789267 0.142679 2.073651
O 2.890542 0.089998 -1.875673
O 2.590055 2.127049 -0.000663
O 0.029650 -0.159690 -2.001063
O -2.702572 -2.335315 0.036770

O -2.883330 -0.310241 -1.941123
O -0.310864 1.955816 -0.103719
O -2.585251 1.941008 -0.021506
C -1.464765 2.444474 -0.057903

CO2-.(H2O)10, solv
E = -953.159531
C 0.125267 -1.303884 -1.604074
O 1.313162 -1.199651 -1.274114
O -0.752357 -0.504346 -1.953458
O 3.701559 0.487538 -1.454401
O 2.082994 2.295258 0.042874
O -0.518639 2.120541 -0.876234
O -1.950591 1.807845 1.304455
O -4.307668 1.166218 -0.037371
O 1.769187 -0.247100 1.374669
O -0.726261 -0.564858 2.503312
O -1.715193 -2.651280 0.863628
O -3.325518 -1.368764 -1.013379
H -1.203976 0.213261 2.166653
H -1.117917 -1.326516 2.025366
H -2.415047 -2.263560 0.290959
H -0.990210 -2.800232 0.241216
H -1.859535 2.602357 1.838931
H -1.370174 1.949943 0.492854
H 0.431541 2.252659 -0.650600
H -0.556186 1.271024 -1.362761
H 2.720994 1.898760 -0.583433
H 1.982882 1.585201 0.703827
H 0.950089 -0.377493 1.908493
H 1.551528 -0.616930 0.496653
H -2.533458 -1.058947 -1.492604
H -3.756113 -0.558714 -0.685534
H -3.545475 1.411653 0.528951
H -4.270649 1.787234 -0.771341
H 2.931271 -0.080438 -1.630869
H 4.138167 0.058284 -0.697362
O 4.445958 -0.807986 1.070663
H 4.490370 -1.766440 1.005985
H 3.537243 -0.625121 1.393891

CO2-.(H2O)10, surf
E = -953.162116
O -1.383262 -1.322955 0.207180
O -1.453563 1.459444 0.220728
O 0.596840 2.357564 -1.534362
O 0.622693 -2.204884 -1.408590
O 1.741417 -0.104670 -2.566984
O 3.593842 0.048420 -0.410118
O 2.620072 -2.200080 0.698733
O 2.340452 2.233729 0.820191
O 0.365052 1.389994 2.319868
O 0.381508 -1.412791 2.409718
H -1.308489 -0.343215 0.176724
H -2.356658 -1.461397 0.075431
H -0.966855 1.857708 -0.525751
H -2.422961 1.439729 0.009061
H 0.912181 1.556656 -1.987919
H 1.227097 2.480685 -0.804716
H 1.829731 -0.260391 -3.511438
H 1.259763 -0.900255 -2.192735
H 1.261524 -2.436906 -0.713903
H -0.198550 -1.956089 -0.916337
H -0.353595 -1.545694 1.777145
H 1.166196 -1.736261 1.937252
H -0.407760 1.502576 1.726710
H 0.408028 0.421448 2.480936
H 1.616379 1.909915 1.443709
H 2.764478 2.975811 1.259982
H 3.117981 0.059025 -1.261663
H 3.261651 0.835335 0.068049
H 3.052862 -1.376087 0.348712
H 3.329020 -2.802977 0.938433
O -4.081195 -1.240322 -0.215386
C -4.545775 -0.102338 -0.352104
O -4.083528 1.045853 -0.317859

CO2-.(H2O)12, incorp
E = -1106.071512
O 3.800608 -0.270175 1.243775
H 4.591725 -0.512249 1.733363
H 3.220987 -1.088971 1.225804
O -0.529132 1.389078 -1.149312
H -0.431093 0.425211 -1.335718
H -1.501054 1.540444 -1.225869
O 2.363952 2.172322 1.617308
H 2.320249 2.472844 0.694395
H 2.899220 1.357517 1.585206
O 2.110874 -2.217553 -1.786796
H 2.591735 -1.373794 -1.895082
H 1.170732 -1.997273 -1.905816
O -3.161214 -1.640774 1.498611
H -3.371328 -1.640183 0.550604
H -3.284203 -0.703342 1.755440
O -0.577552 -1.349186 -1.351824
H -1.543502 -1.355474 -1.525491
H -0.500060 -1.522453 -0.388071
O -0.276810 1.222002 1.652095
H 0.599231 1.604502 1.868092
H -0.356047 1.353124 0.684016
O 1.874317 2.575810 -1.253194
H 1.919949 3.356378 -1.812448
H 0.943114 2.245184 -1.310584
O 2.281612 -2.421891 0.991294
H 1.358261 -2.289880 1.278488
H 2.231758 -2.498984 0.011016

O 3.576964 0.234873 -1.599060
H 3.039636 1.045111 -1.604341
H 3.818027 0.110509 -0.664442
O -3.160502 1.186430 1.863438
H -3.311404 1.425869 0.933297
H -2.212163 1.350713 2.004379
O -0.382422 -1.575009 1.447635
H -1.333173 -1.775560 1.600724
H -0.295805 -0.620859 1.658910
C -3.785722 0.235185 -1.439903
O -3.289007 1.332031 -1.131212
O -3.362996 -0.927894 -1.452315

CO2-.(H2O)12, solv
E = -1106.061709
C -0.876924 -1.437011 1.669041
O -1.566097 -2.206125 1.003871
O -0.849299 -0.193161 1.807633
O 2.699952 -1.459553 0.909749
O 5.161138 -0.628679 -0.070489
O 1.843386 0.597897 2.334859
O 2.064878 2.536830 0.325945
O 3.563934 1.173383 -1.665479
O 1.384870 -0.728515 -1.538392
O -3.474471 1.266958 1.594706
O -2.632027 -0.200981 -2.171360
O -0.806255 -2.390207 -1.895065
O -2.788558 2.761544 -0.813791
O -0.425338 1.174962 -0.636655
O -4.077775 -1.040713 0.057298
H 4.229875 0.610804 -1.229575
H 2.875911 0.549637 -1.950424
H 0.710340 -1.399712 -1.792731
H 1.806949 -1.072737 -0.727368
H 5.600810 -0.203364 0.671650
H 4.370707 -1.058284 0.317331
H 2.390539 -0.707090 1.498743
H 2.345102 -2.263421 1.303936
H 0.881336 0.431623 2.355789
H 1.962719 1.365203 1.731758
H 2.660612 2.172643 -0.362431
H 1.176545 2.411505 -0.042961
H -3.367083 -1.596237 0.423563
H -4.071206 -0.253688 0.638902
H -0.620476 0.746281 0.222692
H 0.253071 0.584602 -1.039444
H -2.607566 0.903293 1.840749
H -3.287287 1.906036 0.882837
H -1.867954 2.451205 -0.869980
H -3.238084 2.251148 -1.498746
H -1.939451 0.293470 -1.704189
H -3.247665 -0.487884 -1.458722
H -1.048921 -2.557742 -0.969343
H -1.493973 -1.764342 -2.198921

CO2-.(H2O)12, surf
E = -1106.059678
O 2.229089 0.338672 -1.375669
O 2.301778 3.085642 -1.586160
O 0.347577 3.403810 0.217345
O 3.606120 -1.819854 -0.290774
O 1.554239 -3.580619 -0.055813
O 2.230488 0.020475 1.504322
O 1.669399 2.482131 2.578259
O 0.470230 -1.977281 2.164855
O -0.252129 -0.253665 -2.542318
O -0.829228 -2.541110 -1.331191
O -1.467129 1.368280 -0.439622
O -1.813695 -1.143762 0.843069
C -4.797173 0.336123 0.168087
H 1.401634 0.153382 -1.863338
H 2.399763 1.296598 -1.507171
H -0.483734 -1.155405 -2.179739
H -0.495020 -0.253852 -3.472357
H -2.802300 -1.087460 0.937046
H -1.575298 -0.243032 0.539576
O -4.176121 1.227910 -0.425382
O -4.480890 -0.666914 0.819033
H -0.375897 2.767804 0.030209
H 0.704762 3.154857 1.090062
H 2.483479 2.866781 2.912925
H 1.906184 1.571791 2.282576
H 1.859728 -4.490148 -0.113392
H 2.349346 -3.004214 -0.196966
H -0.037039 -2.972159 -0.966912
H -1.301365 -2.163579 -0.552770
H 3.289824 -1.130550 -0.909715
H 3.516834 -1.360709 0.561627
H 1.565337 3.264363 -0.937805
H 3.007233 3.695921 -1.357146
H 0.733027 -2.688397 1.559945
H -0.431208 -1.717405 1.876096
H 2.027122 0.196535 0.565150
H 1.567379 -0.649670 1.802681
H -2.460343 1.409252 -0.467258
H -1.184228 0.972845 -1.281482

CO2-.(H2O)14, incorp
E = -1258.976770
O -0.040762 -1.311480 1.613195
H 0.880168 -1.614940 1.812472
H -0.037959 -0.338720 1.707944
O 2.652554 2.159805 -1.560814
H 3.035840 2.714324 -2.246438
H 3.207706 1.336273 -1.529764
O -4.282126 0.026826 1.271420

H -3.749980 -0.775520 1.503791
H -4.470022 -0.077981 0.322911
O -2.748438 -2.215662 1.582369
H -2.753719 -2.495733 0.644362
H -1.813022 -2.006755 1.763614
O -0.011302 1.191527 -1.432640
H 0.877502 1.538944 -1.641791
H 0.038961 0.208395 -1.482342
O -2.639772 2.133215 -1.523079
H -1.699174 1.885637 -1.632537
H -2.724080 2.384847 -0.585731
O 2.516632 2.570326 1.375216
H 1.593835 2.309487 1.545626
H 2.574233 2.628301 0.407906
O -2.899619 2.274259 1.346816
H -3.344532 2.913635 1.910141
H -3.431805 1.424962 1.401295
O 2.573273 -2.077128 1.755397
H 3.142220 -1.291324 1.903838
H 2.730792 -2.286167 0.816751
O -0.003255 -1.562437 -1.142989
H -0.027419 -1.531746 -0.156243
H 0.873413 -1.936229 -1.350503
O -2.688075 -2.517766 -1.250240
H -1.750919 -2.300630 -1.409098
H -3.181033 -1.719125 -1.501494
O -4.209227 -0.046193 -1.627241
H -4.875797 0.045478 -2.313708
H -3.633050 0.762316 -1.684049
O 4.172399 0.228425 1.636866
H 3.649708 1.051342 1.696543
H 4.278299 0.090743 0.677842
O -0.161039 1.511493 1.344510
H -1.052763 1.856607 1.534290
H -0.130719 1.439954 0.366054
C 3.297489 -1.123405 -1.691098
O 3.950124 -0.159414 -1.244563
O 2.819682 -2.140288 -1.186109

CO2-.(H2O)14, solv
E = -1258.963759
C -0.932344 2.071796 -0.354923
O -0.981720 1.117482 -1.162204
O -1.490089 2.341073 0.700385
O -1.009444 -1.550791 -0.227104
O 0.973289 -1.199239 1.670771
O -0.924160 0.353722 3.012034
O -3.088962 -1.127331 1.921130
O -4.222689 1.149088 0.764109
O -3.818188 0.302143 -1.911708
O 1.638947 0.420243 -2.004050
O 2.949200 0.417436 0.443725
O 4.848623 -1.567144 0.514813
O 1.317091 -2.346090 -2.007319
O 2.576680 -3.273145 0.341366
O -3.521959 -2.468839 -1.142055
H 3.444563 -2.835660 0.431445
H 2.019478 -2.838110 1.006308
H 0.409411 -0.689026 2.293431
H 1.653159 -0.571380 1.351809
H 5.426367 -1.470031 -0.247282
H 4.268291 -0.772843 0.505616
H 2.515209 0.458524 -0.440208
H 3.106670 1.350210 0.700608
H 0.699570 0.630529 -1.837285
H 1.644522 -0.549142 -2.174558
H 1.828701 -2.744665 -1.269974
H 0.412195 -2.317324 -1.660508
H -3.441989 1.721483 0.841791
H -4.254417 0.928041 -0.189109
H -1.058770 -0.626446 -0.545668
H -0.276000 -1.535192 0.432754
H -2.891551 0.587453 -1.926569
H -3.780307 -0.666558 -1.800253
H -2.590492 -2.400766 -0.863736
H -4.006378 -2.546325 -0.311968
H -2.464663 -1.347524 1.211282
H -3.604081 -0.369319 1.565129
H -1.042352 1.121541 2.431604
H -1.735484 -0.173817 2.861765
O 3.181849 3.181566 0.812605
H 2.638670 3.565054 1.507980
H 2.703063 3.371333 -0.026114
O 1.725538 3.324810 -1.537072
H 1.933047 2.496004 -1.997576
H 0.805582 3.193303 -1.236569

CO2-.(H2O)14, surf
E = -1258.970330
O 2.177842 0.954722 0.737468
O 1.793341 -1.094137 -1.094297
O 0.321538 -3.087721 0.221849
O -0.393107 -1.958863 2.787111
O 0.701666 0.435975 3.000319
O -1.283774 2.412155 2.327124
O -3.294747 1.101424 1.200696
O -2.988637 -1.599165 1.665335
O 0.534725 3.056395 0.209878
O -0.623808 2.618679 -2.080998
O -3.108656 1.590383 -1.647132
O -2.389608 -2.898849 -0.802952
O -2.393652 -1.044182 -2.692060
O 0.125188 -0.074330 -3.042648
H 3.173137 0.941639 0.688604
H 1.920768 0.245293 0.104683
H -2.882178 -2.092020 0.830719

H -2.150021 -1.742619 2.143150
H -3.790453 2.158348 -2.017601
H -2.246409 2.033174 -1.838568
H -2.401109 -2.216164 -1.534680
H -2.826351 -3.679120 -1.156110
H -0.561522 -3.099094 -0.185943
H 0.167807 -2.757970 1.124849
H -3.231962 0.130646 1.365207
H -3.269479 1.217145 0.234990
H 1.404724 -1.881275 -0.657417
H 2.775835 -1.214644 -1.148945
H 0.786175 -0.473233 -2.432181
H 0.030981 0.845585 -2.748912
H -1.650719 3.026984 2.968581
H -2.059613 1.925160 1.924986
H -0.364460 3.331664 -2.671863
H -0.165066 2.801680 -1.199203
H -2.800196 -0.222666 -2.370975
H -1.459036 -0.780533 -2.888745
H -0.178794 -2.444087 3.589063
H 0.040397 -1.060359 2.882406
H 0.028037 1.128603 2.881265
H 1.348966 0.589778 2.272457
H 1.237134 2.377877 0.369076
H -0.085229 2.954014 0.954661
O 4.837852 0.646778 0.375493
C 5.148608 -0.276223 -0.389649
O 4.523462 -1.107572 -1.058394

CO2-.(H2O)16, incorp
E = -1411.883880
O -2.245511 0.977508 1.637702
H -3.206564 0.902117 1.784278
H -1.917508 0.052101 1.584035
O 1.385369 -1.106442 -1.589641
H 1.623643 -0.159779 -1.608718
H 1.351234 -1.316680 -0.631915
O -4.888093 0.745154 -1.226942
H -4.051800 1.212798 -1.397922
H -4.727845 -0.176009 -1.538088
O -4.204182 -1.901881 -1.734714
H -4.258276 -2.235603 -0.816859
H -3.250217 -1.832856 -1.909262
O -0.042053 2.787226 1.934865
H 0.022527 3.055403 1.000516
H -0.857449 2.257386 1.983016
O -1.429876 -1.624392 1.091197
H -1.430631 -1.520026 0.113071
H -0.496279 -1.796707 1.318607
O 2.082717 1.056343 1.574904
H 1.391391 1.657371 1.933938
H 2.123865 1.307354 0.625443
O 4.159752 -1.962480 -1.528191
H 3.226684 -1.843698 -1.782393
H 4.516324 -1.043173 -1.527833
O -2.142260 1.586197 -1.095663
H -1.424443 2.223393 -1.263532
H -2.148841 1.470683 -0.120888
O -4.196035 -2.396996 1.088063
H -4.558341 -1.537955 1.362167
H -3.245070 -2.334434 1.283695
O -5.005468 0.403732 1.409484
H -5.804268 0.774682 1.794544
H -5.046814 0.585394 0.426390
O 4.823127 0.286747 1.644211
H 4.671411 -0.672536 1.634445
H 3.951316 0.666946 1.855700
O 4.050407 -2.502721 1.077669
H 4.142529 -2.387999 0.088052
H 4.501887 -3.320315 1.304963
O 1.377193 -1.565438 1.213323
H 1.591754 -0.637334 1.472378
H 2.204110 -2.065086 1.343818
O -1.446420 -1.075337 -1.651178
H -0.496601 -1.154090 -1.870964
H -1.630163 -0.112734 -1.603065
O 4.875946 0.693145 -1.184620
H 4.054324 1.188068 -1.339767
H 4.980150 0.684440 -0.211572
C 1.276968 2.524125 -1.542751
O 2.100710 1.660983 -1.167964
O 0.311695 3.059169 -0.996225

CO2-.(H2O)16, solv
E = -1411.868376
C 0.193088 -2.227603 -0.578607
O 0.507102 -2.648504 0.540522
O 0.647531 -1.371574 -1.349387
O 1.780025 1.202216 -0.753498
O 0.311637 2.434284 1.218013
O 2.098898 1.808781 3.291060
O 3.594713 0.465187 1.420466
O 3.361705 -2.265485 1.083682
O 3.669308 -1.952483 -1.725104
O -1.719572 0.352501 -1.769439
O -2.131521 1.178972 0.882544
O -3.515758 -1.054255 1.647115
O -4.056739 -1.312659 -1.107879
O -3.397594 3.611145 0.523578
O -0.338342 2.612961 -2.546074
O -0.860223 4.432609 -0.496165
O 4.536195 0.779719 -1.503621
H -1.784657 4.330160 -0.201218
H -0.345072 3.988532 0.199704
H 0.828607 2.301499 2.039332
H -0.528010 1.934477 1.307909

H -4.170451 3.466179 -0.029639
H -3.053824 2.712511 0.727877
H -1.982671 0.864857 -0.036958
H -2.585757 0.424064 1.327026
H -0.988953 -0.287659 -1.808753
H -1.350566 1.174939 -2.170457
H -0.553139 3.336842 -1.914544
H 0.539893 2.312663 -2.270527
H 2.407623 -2.456695 1.075319
H 3.606575 -2.304365 0.136320
H 1.363234 0.332070 -0.903841
H 1.217702 1.670568 -0.090611
H 2.723547 -1.821609 -1.887610
H 4.056887 -1.058336 -1.794217
H 3.643078 1.161029 -1.452041
H 4.819386 0.776380 -0.578678
H 2.989607 0.696717 0.692604
H 3.573560 -0.520929 1.431261
H 1.835105 1.212706 3.997688
H 2.731936 1.295661 2.734498
H -2.970546 -1.841456 1.809884
H -3.856275 -1.171868 0.734123
H -3.414662 -0.698413 -1.501812
H -3.669628 -2.198277 -1.245197
O -2.597841 -3.773546 -1.039219
H -1.708661 -3.446663 -1.256951
H -2.544487 -3.893868 -0.076484
O -1.813680 -3.457270 1.753242
H -1.677783 -4.053423 2.495100
H -0.916869 -3.213466 1.423844

CO2-.(H2O)16, surf
E = -1411.873036
O 0.373955 -1.941575 -2.190431
O -1.862307 -3.134426 -1.538595
O -0.114796 0.745064 -3.146326
O 1.500874 2.411515 -1.887839
O 2.836409 0.994179 0.009398
O 2.026511 -1.645171 -0.039803
O 0.439637 -2.074156 2.190670
O -2.018840 -3.188820 1.326892
O -3.857737 -1.136359 -1.426478
O -2.708934 1.250092 -2.153488
O -2.597575 2.836841 -0.103798
O -0.078388 3.913640 0.047210
O 1.439083 2.311884 1.947732
O -0.205458 0.629316 3.157083
O -2.794908 1.148430 2.221404
O -4.018281 -1.086405 1.437762
H 0.251565 -1.029938 -2.509430
H 1.029672 -1.889937 -1.453628
H -1.894623 -3.946831 -2.051710
H -0.995626 -2.685185 -1.784614
H -3.226602 -1.870633 -1.557542
H -4.017560 -1.119669 -0.465755
H 1.003952 3.005687 -1.302073
H 2.104145 1.910687 -1.284392
H -3.375781 3.396907 -0.184667
H -2.630150 2.211104 -0.901890
H -1.010275 3.596366 -0.004018
H -0.112469 4.874278 0.062753
H -1.837021 1.051605 -2.549742
H -3.127012 0.381984 -1.939079
H -0.007999 0.679133 4.097302
H 0.438077 1.254376 2.711940
H -2.726571 -2.533700 1.447757
H -1.932834 -3.276325 0.360104
H 2.056583 1.840799 1.336061
H 0.953424 2.931779 1.379686
H -4.821349 -1.175169 1.958604
H -3.568717 -0.255428 1.755668
H 0.077344 0.825121 -4.085557
H 0.513598 1.375573 -2.684850
H -0.403436 -2.503817 1.940456
H 0.196904 -1.193464 2.520758
H -1.897895 0.983854 2.580983
H -2.679939 1.748654 1.462206
H 2.974604 -1.926320 0.026865
H 1.562161 -1.887522 0.793080
H 2.438647 0.092403 -0.010680
H 3.815280 0.801754 0.032076
O 4.725083 -2.053665 0.064649
C 5.509610 -1.098330 0.074483
O 5.389703 0.134847 0.066013

CO2-.(H2O)18, incorp
E = -1564.787908
O 2.335514 1.269321 1.477875
H 2.490384 1.237256 0.507577
H 2.137232 2.206469 1.673278
O -0.077542 0.040069 -1.438721
H -0.064632 -0.932812 -1.600324
H 0.819666 0.378449 -1.609585
O -2.394749 1.602984 -1.450569
H -2.468964 1.638281 -0.474930
H -1.588488 1.070100 -1.600746
O 2.551639 -3.393784 1.597281
H 3.006480 -4.024158 2.163343
H 3.053757 -2.536139 1.676924
O -3.017770 -3.112576 -1.525055
H -2.081955 -3.039812 -1.772732
H -3.418981 -2.235866 -1.698262
O -4.303083 -0.687115 1.434381
H -5.088181 -0.816446 1.974184
H -3.818878 -1.561669 1.441064
O -0.121462 0.017746 1.327770

H 0.730509 0.447888 1.562453
H -0.123913 0.043521 0.342527
O -0.220437 -2.730982 1.418700
H 0.662840 -3.082358 1.633533
H -0.153064 -1.758352 1.544078
O -3.062931 -3.042300 1.245318
H -3.078408 -3.196699 0.271644
H -2.119107 -3.073087 1.480849
O -2.456921 1.485317 1.411965
H -3.178425 0.848102 1.570707
H -1.632529 0.969881 1.546182
O -1.220953 4.101740 1.361164
H -1.759614 3.312669 1.545133
H -1.309360 4.246692 0.388486
O -0.118713 -2.702836 -1.422025
H 0.789216 -3.028095 -1.601811
H -0.207147 -2.798612 -0.450286
O 1.419465 3.910173 1.579653
H 1.723869 4.667819 2.087230
H 0.419104 3.959196 1.576315
O -4.278439 -0.580971 -1.517954
H -3.721056 0.203351 -1.680898
H -4.456267 -0.547967 -0.563332
O 3.922064 -1.102932 1.567035
H 3.464809 -0.284938 1.830257
H 4.088094 -0.978624 0.608936
O -1.213745 4.239699 -1.397462
H -0.265968 4.072791 -1.567807
H -1.669717 3.410302 -1.631135
O 1.603382 3.770570 -1.362612
H 2.006440 2.909278 -1.560729
H 1.701775 3.878203 -0.402295
O 2.605021 -3.392758 -1.344542
H 3.150796 -2.600782 -1.485063
H 2.653328 -3.549818 -0.386183
C 3.425214 0.175317 -1.760635
O 2.630415 1.026102 -1.304739
O 4.013630 -0.780708 -1.254309

CO2-.(H2O)18, solv
E = -1564.765069
C -0.827238 1.632796 -0.482457
O -0.772190 0.682390 -1.278699
O -1.293218 1.790803 0.649611
O 0.585083 4.522965 -0.952323
O 3.182717 3.333276 -1.104932
O 2.952378 2.765053 1.646109
O 3.080111 0.162284 0.828474
O 2.174486 0.646417 -1.790291
O 0.191385 3.783917 1.837403
O 5.517101 -1.064763 0.362674
O 2.355417 -1.973110 -2.625170
O -0.147701 -2.080690 -0.688399
O 1.857289 -2.293260 1.156089
O 0.167491 -2.920119 3.297269
O 3.908803 -3.178512 -0.664152
O -1.945272 -2.562431 1.572226
O -3.365238 -0.155593 1.242022
O -3.427285 -0.567937 -1.593970
O -2.608166 -3.236651 -1.490370
H 4.611939 -2.564188 -0.379909
H 3.261858 -3.140960 0.061572
H 1.410397 -2.517367 1.998656
H 2.250844 -1.399167 1.252947
H 6.061408 -0.478926 -0.171019
H 4.723337 -0.536439 0.602435
H 2.745758 0.350176 -0.075990
H 3.027502 1.031882 1.292872
H 1.209551 0.753548 -1.810740
H 2.337805 -0.230719 -2.212351
H 2.975379 -2.444680 -2.021362
H 1.479484 -2.270639 -2.343328
H -2.697731 0.554939 1.245176
H -3.524207 -0.305224 0.289055
H -0.287328 -1.126165 -0.831137
H 0.619541 -2.165008 -0.069518
H -2.573832 -0.120512 -1.724705
H -3.240473 -1.528023 -1.686529
H -1.659921 -3.052125 -1.359510
H -2.899019 -3.542439 -0.621683
H -1.367395 -2.384084 0.808548
H -2.504798 -1.759235 1.631990
H 0.087662 -2.375044 4.084958
H -0.683018 -2.816486 2.810526
H 2.064015 3.105306 1.841360
H 3.143442 3.074305 0.734481
H 2.991107 2.471381 -1.510700
H 2.359736 3.846702 -1.213406
H 0.034203 3.755428 -1.175540
H 0.493134 4.572262 0.014045
H -0.237053 4.204756 2.588379
H -0.418474 3.074941 1.530712
H -5.101298 0.215848 -1.717785
O -5.967068 0.653551 -1.606581
H -5.877832 1.502160 -2.049435
H -5.325249 0.540644 1.544303
O -6.227393 0.832807 1.347723
H -6.276890 0.783216 0.379665

CO2-.(H2O)18, surf
E = -1564.773820
O 2.916824 2.610590 -0.803412
O 2.650308 2.170508 1.776492
O 4.082643 -0.162154 2.333707
O 0.385313 2.147174 -2.041384
O -1.743168 3.424831 -1.101410

O 0.006451 -0.590480 -1.554698
O 2.252629 -1.611016 -2.468898
O 0.145124 -0.868591 1.183923
O 2.360235 -2.442848 1.935667
O 3.583949 -2.991540 -0.372728
O -0.032956 1.582015 2.559077
O -2.230404 2.721675 1.609341
O 4.204590 0.411159 -1.985313
O 5.495097 -0.891200 -0.081702
O -2.472103 -1.178528 -2.274891
O -3.627490 1.254446 -1.568316
O -2.551679 -1.650817 1.774808
O -4.015352 0.612506 1.214069
H 3.288448 3.492363 -0.900332
H 2.811171 2.458575 0.183719
H 4.371598 -2.421482 -0.305095
H 3.082133 -2.633717 -1.131947
H 0.026517 -0.680051 -0.575653
H -0.912077 -0.880950 -1.838789
H 3.142179 1.361665 2.039059
H 1.727293 2.042405 2.066888
H -0.852226 2.027212 2.186900
H -0.198407 1.502631 3.503959
H 0.123358 -0.036614 1.698764
H 0.901485 -1.394694 1.505776
H 2.163068 -3.234546 2.445062
H 2.769392 -2.744049 1.079050
H -3.002697 -1.777395 -1.711761
H -2.914123 -0.308056 -2.164400
H -3.045530 2.028979 -1.514936
H -3.812753 1.023019 -0.636427
H -1.625251 -1.562385 1.497787
H -3.026950 -2.099565 1.040788
H 1.248274 2.330130 -1.628435
H 0.230704 1.184272 -1.924244
H -1.851204 4.258542 -1.566923
H -0.914228 2.999516 -1.457045
H -4.857542 0.136289 1.043720
H -3.419442 -0.124849 1.487528
H 1.356125 -1.273099 -2.165742
H 2.106492 -2.003278 -3.335251
H 4.671879 -0.354617 1.582851
H 3.506462 -0.944069 2.389204
H 3.755043 1.206511 -1.635134
H 3.499323 -0.199207 -2.275579
H 5.080222 -0.343870 -0.808842
H 6.444245 -0.881102 -0.234177
H -2.977046 2.089822 1.540167
H -2.093677 3.066013 0.707372
O -4.123364 -2.525906 -0.365081
C -5.318936 -2.184035 -0.238931
O -5.897728 -1.297591 0.386462

CO2-.(H2O)20, incorp
E = -1717.685397
O -0.138287 0.098276 -1.366345
H -0.704269 0.893079 -1.506582
H -0.084300 0.017492 -0.387433
O -0.017603 -0.223606 1.434538
H -0.788801 -0.819493 1.578006
H 0.760098 -0.826553 1.512163
O -2.049850 -2.086284 1.392168
H -2.964955 -1.858992 1.633066
H -2.077274 -2.150158 0.412246
O -1.715711 2.360908 -1.287453
H -1.682870 2.410345 -0.305636
H -2.656807 2.198552 -1.492511
O 1.773988 -2.176936 -1.532631
H 1.238361 -1.407341 -1.779540
H 1.167324 -2.955391 -1.620203
O 2.115790 2.072004 1.398357
H 1.490809 1.391430 1.684161
H 1.626270 2.929173 1.503491
O 2.175277 1.980122 -1.427482
H 2.201608 1.995500 -0.446921
H 1.525155 1.286110 -1.627578
O 1.966380 -2.083621 1.243860
H 2.900750 -1.941414 1.499457
H 1.972866 -2.130452 0.261530
O -1.583044 2.283877 1.500965
H -1.069233 1.481783 1.687135
H -2.527873 2.062728 1.685047
O -2.070556 -2.043265 -1.420537
H -1.485949 -1.279498 -1.588261
H -2.985867 -1.737978 -1.563415
O -4.444525 1.658221 -1.389975
H -5.138987 1.969777 -1.978367
H -4.549810 0.671483 -1.339255
O -0.097619 -4.156768 -1.422171
H -0.920217 -3.662900 -1.574489
H -0.097480 -4.336500 -0.458654
O 0.509473 4.259695 1.392931
H -0.337638 3.821324 1.580511
H 0.476063 4.451994 0.431795
O 4.940403 1.119753 -1.466444
H 4.051892 1.453089 -1.682082
H 4.887869 0.154199 -1.616467
O 4.667103 -1.492732 1.500239
H 5.322380 -1.836315 2.114013
H 4.761632 -0.493839 1.502294
O 4.841672 1.132768 1.301052
H 3.987506 1.563899 1.480827
H 4.978684 1.237188 0.329645
O 4.634122 -1.704162 -1.443208
H 4.739015 -1.813787 -0.483934
H 3.715418 -1.965844 -1.626841

O -4.242302 1.659524 1.540118
H -4.442040 0.703338 1.583803
H -4.468220 1.888662 0.623703
O -0.027005 -4.176512 1.417214
H -0.798510 -3.635215 1.652876
H 0.735697 -3.589460 1.560633
O 0.416244 4.304762 -1.439764
H 1.114639 3.649194 -1.617325
H -0.406151 3.815648 -1.610330
C -5.065214 -1.460498 0.012791
O -4.743427 -1.011223 -1.106531
O -4.794960 -1.130035 1.170187

CO2-.(H2O)20, solv
E = -1717.666724
C 1.178305 0.033679 1.214659
O 1.062042 0.644759 2.280793
O 0.923622 0.293137 0.029085
O 3.095017 -2.430467 0.604685
O 0.657336 -3.979490 0.418436
O -1.233804 -3.105699 2.328720
O -2.483747 -1.864121 0.242102
O -0.147274 -1.887730 -1.500969
O -0.021064 -1.164459 4.137400
O -4.636458 -2.833251 -1.191511
O -1.547729 -0.474819 -3.365211
O -1.054036 1.785283 -1.402299
O -3.312374 0.819317 -0.258784
O -3.164144 2.673653 1.764791
O -4.247883 -0.445737 -2.685762
O -1.035068 4.103937 0.426352
O 1.629680 3.512766 1.351525
O 5.493349 -1.075958 0.316710
O 4.578308 0.197975 -1.907927
O 2.682394 2.271417 -1.124641
O 0.690799 3.649387 -2.605190
H -4.517476 -1.303969 -2.307418
H -4.198472 0.145428 -1.915418
H -3.399101 1.440923 0.504272
H -3.071220 -0.056582 0.103662
H -4.437575 -3.721022 -1.501730
H -3.899549 -2.594580 -0.586309
H -1.720190 -1.924927 -0.364421
H -2.156665 -2.272381 1.080698
H 0.280519 -1.143474 -1.040145
H -0.640797 -1.479815 -2.257078
H -2.522704 -0.527334 -3.234170
H -1.319511 0.414261 -3.055081
H 1.528346 2.688346 1.850320
H 2.151923 3.255637 0.570871
H -0.405477 1.301886 -0.858498
H -1.931851 1.466580 -1.065967
H 2.102454 1.547689 -0.833315
H 2.135844 2.780971 -1.757273
H -0.052601 3.057057 -2.387382
H 0.461805 4.484552 -2.182689
H -1.049491 3.407153 -0.250211
H -0.145151 4.028201 0.822236
H -2.716472 2.262272 2.526632
H -2.514492 3.317176 1.421888
H -0.815094 -2.514611 2.976313
H -0.507627 -3.492172 1.797936
H 0.371539 -3.480990 -0.364629
H 1.593673 -3.747298 0.525569
H 2.538511 -1.709955 0.948373
H 4.009051 -2.072309 0.637047
H 0.507586 -1.442949 4.891037
H 0.558047 -0.597163 3.591140
H 4.046000 0.998001 -1.750750
H 3.947748 -0.480701 -2.221398
H 5.871976 -0.450676 0.940342
H 5.249250 -0.548966 -0.486007
O 2.857187 -2.031144 -2.249631
H 1.904141 -2.052508 -2.412943
H 2.937457 -2.294957 -1.313924
H -0.621153 1.469006 3.166108
O -1.433482 1.473114 3.694264
H -1.444401 0.587188 4.080857

CO2-.(H2O)20, surf
E = -1717.674826
O 4.698894 0.193300 -2.557361
O 3.317036 -2.036118 -2.170031
O 0.923600 -1.332194 -0.974458
O 0.692048 1.501718 -1.028224
O 3.287630 2.370740 -1.339194
O -1.372579 2.653652 -2.340067
O -0.158189 1.543098 1.584668
O -1.484509 -0.814230 1.764183
O 2.193217 -1.701182 1.441475
O 0.262819 -2.444497 3.295345
O 2.409220 0.890367 2.493253
O 4.467356 1.950480 1.239755
O -1.731585 -1.824665 -0.754950
O -2.793707 0.133090 -2.329470
O -2.424322 3.198993 1.819984
O -3.586483 3.189024 -0.577844
O 5.916325 -0.143312 -0.006286
O 4.684736 -2.496374 0.354551
O -4.770041 0.623141 -0.370424
O -3.836493 0.656231 2.306060
H 4.142562 -0.636130 -2.493914
H 5.054622 0.218305 -3.450358
H 5.687752 0.046591 -0.935145
H 5.500997 0.578100 0.506029
H 3.748225 -2.426801 -1.391551

H 2.389325 -1.873590 -1.890172
H 0.502061 -2.019439 4.124188
H 1.029575 -2.307084 2.701590
H -2.866413 3.252443 0.925730
H -2.581478 4.041727 2.254544
H -3.094815 0.024051 2.255309
H -3.411177 1.525552 2.360320
H 5.284841 -3.186512 0.650997
H 5.211989 -1.654390 0.296765
H -2.885479 3.075856 -1.243756
H -4.147186 2.388728 -0.636084
H -5.186440 -0.249762 -0.581990
H -4.518816 0.555534 0.574589
H 3.680793 1.588981 1.748544
H 4.877917 2.610997 1.805361
H 0.006131 -1.703966 -0.977474
H 0.808995 -0.364744 -1.088857
H 2.360158 -0.037943 2.180699
H 1.524088 1.260057 2.295649
H -1.626930 -1.222524 0.870534
H -1.026759 -1.477167 2.316776
H 3.729034 2.373820 -0.470914
H 3.757976 1.690431 -1.854450
H 1.603046 1.865587 -1.123726
H 0.434284 1.596805 -0.081355
H -0.839610 2.225926 1.742931
H -0.635400 0.676213 1.663977
H -2.057196 -1.127261 -1.373213
H -2.473295 -2.493495 -0.752300
H 1.693445 -1.592904 0.595488
H 3.050501 -2.090201 1.187078
H -1.087309 3.126871 -3.126433
H -0.556694 2.292710 -1.908385
H -3.514426 0.334958 -1.697853
H -2.326591 0.970151 -2.471671
O -5.644138 -1.922989 -0.881251
C -5.072867 -3.017692 -0.861851
O -3.901474 -3.407063 -0.761760

Cartesian coordinates (in Å) and energies (in Hartree) of the 27 different geometries of $CO_2^{\bullet-}(H_2O)_{10}$ sampled from the MD run at 300 K and optimized at B3LYP/6-311++G level of theory. Labels (MD10-1 – MD10-27) are arranged in ascending order of electronic energy with zero point correction.**

MD10-1

H	0.16887200	-1.46143100	0.76826900
H	1.19830600	-2.32934200	0.00628700
H	3.87276700	-2.41708000	-0.23512000
H	3.10376300	-1.16286100	-0.79099700
H	-0.09701900	0.66830000	1.24987000
H	-0.98760800	-0.18991700	2.16630700
H	-2.94613800	0.46633600	1.43615600
H	-2.88172200	-1.07286800	1.40895100
H	1.84272600	0.01614600	2.29620700
H	2.98883600	-0.62040800	1.49476600
H	1.96898800	0.01656200	-2.17223100
H	2.88586700	0.89714100	-1.30122900
H	1.64314800	2.32237700	-0.01198300
H	2.71978500	1.58280300	0.81385600
H	0.06964100	-0.90932800	-1.37941200
H	-0.07316100	0.62113200	-1.41606000
H	-2.85857300	-1.73498000	-0.73122500
H	-1.76519700	-2.55096900	-0.01696600
H	-2.95660000	0.48238600	-1.36531000
H	-1.95619200	-0.26846600	-2.24038700
O	0.25086400	-2.11158800	0.03326700
O	3.11157900	-1.85461900	-0.06786800
O	-0.05072000	-0.07631600	1.88367600
O	-2.79895200	-0.30289400	2.01080200
O	2.78392800	0.13053400	2.07677700
O	2.89500800	0.09387800	-1.87168400
O	2.59557700	2.12491900	0.00987900

O	0.03243300	-0.15432600	-2.00391100
O	-2.70895100	-2.33297400	0.03038300
O	-2.88173900	-0.30010100	-1.93955500
O	-0.30277900	1.95629300	-0.09832400
O	-2.57724700	1.94683200	-0.02139800
C	-1.45541600	2.44789300	-0.05268600

-953.4350 -953.16747 -953.1399 -953.2232

MD10-2

H	1.55485700	-2.26156100	2.71318600
H	1.17305300	-1.96188300	1.23629600
H	0.40107700	2.55353100	0.85004700
H	1.55595700	3.59594400	0.67613100
H	-1.74566700	2.14472100	0.71496600
H	-1.14462700	1.46999200	1.97306900
H	-2.57507800	-1.57882300	0.01260200
H	-3.15405100	-0.17543600	0.21368000
H	-0.40689800	-0.66193900	2.52622100
H	-1.88812400	-0.60505700	2.05407100
H	-3.49599100	1.99390900	-1.16342000
H	-2.19528600	1.14021400	-1.41816700
H	-1.09252000	-0.49502100	-2.18605500
H	-0.29306600	0.83135300	-2.44014300
H	2.53479200	-0.11362700	1.66235000
H	2.52173600	1.29789900	1.03366200
H	3.13051100	-0.27089200	-0.54402800
H	2.21968000	-1.30314200	-1.22618200
H	1.92329700	0.79532600	-2.07037900
H	1.32250000	2.02832800	-1.37204600
O	1.30166300	-1.54426200	2.12473200
O	1.31859200	2.68666800	0.47367000
O	-1.12144400	2.30370200	1.44773600
O	-3.05977700	-1.03604200	0.65852800
O	-1.24381900	-0.18551700	2.66222200
O	-2.80955600	1.50127200	-0.70562600
O	-1.19573100	0.43452200	-2.47167700
O	3.09705300	0.52860200	1.19614400
O	2.88492500	-0.61859300	-1.42385200
O	1.31568800	1.55326900	-2.22062400
O	-1.18371200	-2.18229500	-1.30460400
O	0.88777300	-2.46473300	-0.38709900
C	-0.31323300	-2.70609800	-0.60704400

-953.4325 -953.16657 -953.1385 -953.2234

MD10-3

H	-2.43116500	3.07455100	1.01043200
H	-2.01729100	2.39133500	-0.30326600
H	-1.37122400	-1.78192600	2.96988500
H	-0.35366600	-0.86695400	2.21916900
H	1.80987500	-0.53405800	1.93485200
H	1.11906600	0.84561800	2.06439700

H	2.64924100	1.17902600	-1.19692800
H	3.44052600	0.47109700	-0.09706000
H	0.66577200	2.41576200	0.44632300
H	2.15482300	2.22344500	0.68995400
H	3.74265900	-1.92957500	0.91897600
H	2.51409000	-1.67811600	-0.03388300
H	1.32771400	-1.29365000	-1.72160400
H	0.71363200	-2.50632700	-0.96638800
H	-3.30744400	0.79624800	0.67584000
H	-2.70901700	-0.50391500	1.27714500
H	-3.00858200	-0.82695400	-0.91686000
H	-1.93598100	-0.50630200	-1.97507800
H	-1.51233800	-2.48223900	-0.89140800
H	-0.97541100	-2.54447300	0.54624400
O	-2.78746600	2.54854300	0.28843200
O	-1.19747100	-1.38936100	2.10955800
O	1.10334100	-0.07177900	2.42250700
O	3.33614600	1.33911700	-0.52317600
O	1.32211500	2.41074200	1.16823200
O	3.06263600	-1.28360600	0.70979600
O	1.56897000	-2.13204900	-1.28556400
O	-3.45027300	-0.16762500	0.74347000
O	-2.56125800	-1.20008200	-1.70760000
O	-0.87580900	-2.96853600	-0.32396400
O	0.96440100	0.56673100	-2.04996600
O	-0.45893100	2.05631000	-1.07684900
C	-0.12108000	1.06502900	-1.74162500

-953.4312 -953.16528 -953.1370 -953.2227

MD10-4

H	3.63940400	-2.63228900	-0.43448800
H	2.16088500	-2.34819800	-0.09941400
H	1.15525200	2.03064800	2.89534000
H	0.17682300	1.05012400	2.17776100
H	-1.96855500	0.65841100	1.81533000
H	-1.26950300	-0.68560600	2.13660900
H	-2.55205000	-1.37131400	-1.18479500
H	-3.43976700	-0.59291400	-0.21310200
H	-0.66097400	-2.41008200	0.76766500
H	-2.17180300	-2.21281000	0.81914600
H	-3.88030800	1.88049600	0.54770900
H	-2.58541400	1.58326200	-0.29660500
H	-1.27702200	1.08106100	-1.86330300
H	-0.75056500	2.37996600	-1.19011900
H	3.31168500	-0.61783400	0.85434000
H	2.59845800	0.66056300	1.39495700
H	2.99757000	0.86309300	-0.79545500
H	1.98014000	0.39576500	-1.85274500
H	1.46192700	2.45244200	-0.99316800
H	0.84864600	2.59929800	0.40742800
O	3.06268500	-2.39078900	0.29580200
O	1.02065700	1.57207200	2.06094700

O	-1.28270100	0.26774300	2.38699100
O	-3.28340400	-1.49140300	-0.55075000
O	-1.39560000	-2.33066300	1.40263800
O	-3.16843300	1.24301200	0.44627600
O	-1.57051300	1.94857800	-1.52893200
O	3.38885300	0.35389400	0.91631500
O	2.58709900	1.12689200	-1.64854100
O	0.78112400	2.95441900	-0.49655800
O	-0.83850200	-0.79886100	-1.97926400
O	0.55166000	-2.13031600	-0.75742700
C	0.23362900	-1.22678300	-1.54551100

-953.4308 -953.16483 -953.1366 -953.2222

MD10-5

H	3.63748500	-2.39313200	-1.15444000
H	2.25888200	-2.06667000	-0.54292300
H	0.69563100	-0.35802500	0.90629900
H	-0.07484400	-0.01459600	2.18725500
H	-2.34499400	0.10967700	1.99470800
H	-1.82669300	-1.33798400	1.98667900
H	-2.45384000	-0.80905700	-1.69789500
H	-3.43215800	-0.33976500	-0.61578500
H	-0.87166100	-2.41740900	0.18598700
H	-2.37371500	-2.26735500	-0.01463900
H	-3.99691700	1.76951600	0.80019100
H	-2.60951900	1.69918600	0.07451500
H	-1.20228500	1.69724500	-1.54103200
H	-0.66706900	2.63714700	-0.43276900
H	3.60787300	-0.84128400	0.80862900
H	2.77064800	0.09425300	1.69818000
H	3.32751900	1.16137000	-0.20682300
H	2.48072200	1.09271300	-1.49232800
H	1.58952500	2.63917200	-0.01761100
H	0.76384200	1.97079800	1.08027700
O	3.17988000	-2.32219200	-0.31200500
O	0.76933800	0.17428100	1.71654000
O	-1.79630700	-0.50806600	2.50822300
O	-3.24717700	-1.08997600	-1.20748800
O	-1.71748700	-2.62024400	0.61982800
O	-3.26771300	1.17240600	0.61135900
O	-1.48463100	2.40261200	-0.93592700
O	3.61820800	0.04116700	1.22453900
O	2.98992400	1.70981100	-0.94926500
O	0.79556800	2.78063900	0.53377400
O	-0.65195300	-0.13644900	-2.20970300
O	0.61991500	-1.40502700	-0.80326700
C	0.37684000	-0.60291500	-1.72989000

-953.4295 -953.16399 -953.1352 -953.2225

MD10-6

H	-1.42985500	-1.69142300	-2.29565200
H	-1.82272000	-2.31822300	-0.97885000
H	-1.95263700	3.40608600	-0.82949900
H	-0.80371800	2.37312800	-1.08941700
H	1.38724700	2.03112300	-1.42257700
H	0.47470100	0.96539400	-2.05097900
H	2.84448100	-1.46611000	-0.29604000
H	3.24133700	-0.12152500	-0.92040200
H	0.17787200	-1.10226100	-1.04357200
H	1.42298800	-1.02589800	-1.92778000
H	3.59966500	2.34627300	-0.09929600
H	2.49135600	1.47410600	0.59847300
H	1.78699600	-0.06263300	1.88035900
H	0.87461400	1.19856700	2.07016900
H	-3.14083900	-0.42685900	-1.11089900
H	-2.89759800	1.04605100	-0.67381200
H	-2.97147500	-0.26897100	1.15769500
H	-1.80508300	-1.16232300	1.59251300
H	-1.40132800	0.95563400	2.21386800
H	-1.08008200	2.13239700	1.28326300
O	-2.21220400	-1.97895700	-1.80037700
O	-1.62759400	2.54449200	-0.55375900
O	0.56573700	1.93867100	-1.93988100
O	3.14526000	-1.06582800	-1.13299800
O	0.46217300	-0.83445200	-1.94368200
O	2.89122800	1.72503500	-0.28884300
O	1.81266100	0.90729800	1.96429000
O	-3.50337400	0.29348300	-0.56124000
O	-2.44943700	-0.52596900	1.94502400
O	-0.81233500	1.73817200	2.13088500
O	1.76604500	-1.96105100	1.28367300
O	-0.36744300	-2.03501400	0.49434800
C	0.60638800	-2.37492800	1.19381800

-953.4299 -953.16365 -953.1355 -953.2205

MD10-7

H	3.69822900	-2.56855200	-1.12655500
H	2.31911100	-2.00386100	-0.74028600
H	0.77683600	-0.14985500	0.68611500
H	-0.05089600	-0.03009500	1.99745800
H	-2.28085600	0.31833800	1.91180400
H	-2.04935200	-1.19920900	2.02783600
H	-2.63375500	-0.73525900	-1.64317800
H	-3.48970200	-0.08288800	-0.55276100
H	-1.75323600	-3.06630700	0.52797500
H	-2.90398200	-2.11495700	0.13540600
H	-3.75814900	2.13010100	0.77093700
H	-2.39580300	1.87813000	0.02719500
H	-0.98477900	1.61041600	-1.50743400

H	-0.37393400	2.63422100	-0.49867600
H	3.57310400	-1.18775900	0.97517400
H	2.74234700	-0.20097100	1.80712900
H	3.52110600	0.87833400	-0.01155000
H	2.53507300	0.75716700	-1.16695300
H	1.88776900	2.56414000	0.00623700
H	0.96715900	2.04803000	1.09808100
O	3.08571400	-2.50774400	-0.38800700
O	0.82186100	0.18780200	1.59825600
O	-1.77929900	-0.34511100	2.41807200
O	-3.41039500	-0.89954500	-1.07858000
O	-2.53924800	-2.64099900	0.88528100
O	-3.11733800	1.44220300	0.57110900
O	-1.20380600	2.40032400	-0.97748900
O	3.64081400	-0.34658800	1.46138300
O	3.18531700	1.36970600	-0.78865500
O	1.09001300	2.81810800	0.51050900
O	-0.85167600	-0.19608200	-2.20600900
O	1.09248100	-0.77199500	-1.17237400
C	0.08970100	-0.92663300	-1.89865500

-953.4286 -953.16334 -953.1344 -953.2225

MD10-8

H	-1.11711800	2.39322200	-0.56069500
H	-0.32253800	1.30692300	0.19790700
H	0.07023200	-0.61402200	-2.18789600
H	0.35876400	0.86548000	-1.98358000
H	-3.23613800	3.38763900	0.15865000
H	-3.23616100	1.85545800	-0.06541600
H	-3.70727900	-2.87371600	-0.14312100
H	-2.55771300	-2.13461500	-0.85872800
H	-2.62606800	0.19807400	1.20798700
H	-3.65246000	-0.44154100	0.25206400
H	-0.78880600	-1.16166100	-0.12450500
H	-0.03762900	-2.38911300	-0.64463100
H	1.54919500	-2.43687500	0.87793700
H	2.17742300	-2.35841300	-0.52189300
H	1.32939200	2.82286100	-0.17118200
H	2.64680300	3.18786900	-0.87987000
H	2.99703500	1.46798600	0.73782700
H	2.66202500	0.14465000	1.47713500
H	3.26491400	-0.43581600	-0.65791600
H	2.32356900	-0.57935300	-1.87985300
O	-0.22282000	1.99868400	-0.48795000
O	0.63933400	0.10301200	-2.52013500
O	-2.93394100	2.71346600	-0.45636000
O	-3.52754700	-2.10326200	-0.68947100
O	-3.49304500	0.35922000	0.78784700
O	-0.80705500	-1.80947300	-0.85802300
O	1.56239200	-2.89977200	0.02504900
O	2.25994200	3.09112400	-0.00448900
O	3.33412000	0.57159700	0.91976600

O	3.05275700	-1.02723900	-1.40852900
O	1.14166200	-0.88691200	2.30356200
O	-0.76606500	-0.04998000	1.38515000
C	-0.00255400	-0.44812700	2.29974000

-953.4279 -953.16241 -953.1335 -953.2214

MD10-9

H	0.59241100	-4.02441300	0.75899500
H	0.35693100	-3.16425000	-0.49339400
H	2.27395300	0.47719300	2.93570300
H	0.93927900	0.34452600	2.13027900
H	-0.99482900	1.13924300	1.55229100
H	-1.25475700	-0.29456600	2.06634900
H	-3.48065200	1.20305000	0.14526600
H	-3.34240400	0.11603200	-0.89181500
H	-1.96950400	-1.89812300	0.60577400
H	-3.11466800	-0.98593400	1.03617100
H	-1.37703300	1.49433100	-0.67484700
H	-0.92541000	2.86051700	-0.08772500
H	0.58131400	4.36918400	-1.25905900
H	1.24698500	3.34511000	-0.29817700
H	2.40380800	-2.44857600	0.22686200
H	2.66113000	-1.09897300	0.95239900
H	2.66333200	-0.68590100	-1.23280600
H	1.41307800	-0.29779100	-2.04663300
H	2.43276000	1.46957700	-0.85617500
H	2.34810600	1.62918600	0.67012100
O	1.03846900	-3.70144300	-0.02938000
O	1.93662900	0.32861200	2.04777200
O	-0.70400700	0.50008600	2.23628100
O	-3.90246400	0.37790700	-0.13833500
O	-2.42330900	-1.56849100	1.40380300
O	-1.53506500	2.10869700	0.06449900
O	0.48888900	3.98102800	-0.38469600
O	3.01535100	-1.68789700	0.26322200
O	2.33283400	-0.02919000	-1.88500900
O	2.45650000	2.14960100	-0.14619400
O	-1.56740400	-0.04710400	-1.82263700
O	-0.94676000	-2.09135300	-1.03181000
C	-0.82852200	-1.01884300	-1.64328400

-953.4274 -953.16204 -953.1333 -953.2208

MD10-10

H	3.12809600	3.20991900	0.01109200
H	2.09774800	2.38256900	-0.78008700
H	2.11014200	-1.70110500	-1.65392900
H	2.24096400	-2.68572900	-0.51603100
H	-0.50598400	-0.78420700	-2.14884800
H	0.48591400	0.21859000	-1.50970800
H	-2.52971400	2.02140200	-0.49726200
H	-2.50086500	1.13341600	-1.74462700

H	0.08387600	1.77289100	0.12738900
H	-0.45710300	2.19779000	-1.24428000
H	-3.05152200	-1.23371700	-2.62843900
H	-2.55405500	-1.24606100	-1.13874600
H	-2.73409100	-1.25680400	1.02304200
H	-1.65838600	-2.26107200	0.52922300
H	3.46235900	0.78398900	0.23968600
H	3.43718700	-0.75677000	0.04325800
H	2.07798100	-0.38261600	1.87630400
H	0.62951900	0.04469200	2.27287800
H	0.55480900	-1.89635900	1.23066400
H	0.26157500	-1.80208100	-0.28030800
O	3.05885900	2.47234700	-0.60179500
O	2.78274100	-2.17655200	-1.13675300
O	0.41575600	-0.69994800	-1.84674700
O	-2.36738200	2.05570300	-1.45916200
O	0.37315300	1.92464000	-0.80509800
O	-2.44222800	-0.79523700	-2.02790200
O	-2.57684000	-1.95564600	0.35991200
O	3.53352600	-0.06101500	0.71937600
O	1.26747200	-0.69031000	2.33108700
O	0.19109300	-2.41634800	0.47385200
O	-2.71119600	0.38586800	2.02122300
O	-0.67221400	1.35574800	1.65749700
C	-1.90006000	1.17454500	1.55080100

-953.4260 -953.16008 -953.1317 -953.2177

MD10-11

H	-2.66501200	1.63196700	-0.56097000
H	-3.22854900	0.32376400	-1.20891300
H	-3.87976100	-1.99321200	-1.68837600
H	-3.30576900	-1.87219900	-0.25915600
H	-0.63836900	-0.00342200	0.77732400
H	-0.05762500	-0.37195600	2.14173600
H	2.30326000	-0.79730800	1.68950500
H	1.29860800	-1.90842800	1.38866700
H	-1.80839700	-1.72059900	1.37389500
H	-1.30591700	-2.86447900	0.49441300
H	0.24456400	-1.68477200	-0.69025600
H	1.40896200	-2.67605000	-0.76959200
H	3.25074700	-1.26168800	-0.88355000
H	3.03077800	-1.65859000	-2.34958800
H	-1.03569600	0.46284000	-1.32588200
H	0.52829600	0.67472900	-1.04871100
H	-1.32041500	2.98275800	0.77330700
H	-1.75063400	1.71275100	1.53754500
H	0.90923700	3.17466500	-0.16646000
H	0.00190000	4.20577500	-0.85529200
O	-2.68558100	1.11351900	-1.39254500
O	-3.91437000	-1.43901300	-0.90371000
O	-0.89503800	-0.09295300	1.72004800
O	1.60998800	-1.33142100	2.11037400

O	-2.11810300	-2.51971500	0.89957300
O	0.54631500	-2.54758900	-0.33504000
O	3.11826100	-2.03918100	-1.47034000
O	-0.20517000	0.02584600	-1.02066600
O	-2.10582300	2.46276900	1.03045100
O	0.20720900	3.83869000	0.00949700
O	1.94339200	1.78030100	-0.66409300
O	3.33732700	0.20247200	0.20897800
C	2.88542300	1.33561000	0.01502200

-953.4246 -953.15952 -953.1304 -953.2189

MD10-12

H	4.42927200	-1.95727400	-0.56259700
H	2.96724500	-2.05202000	-0.10941000
H	1.13614000	1.49617200	2.08800300
H	-0.38784100	1.33710800	2.27879900
H	-2.05501300	-0.19115100	3.27716000
H	-1.42110300	-0.89160300	2.03101200
H	-3.15806700	-1.28499500	-0.59737400
H	-2.02289500	-1.84481300	-1.47226500
H	-0.18219300	-2.19688000	0.77352200
H	-1.68647100	-2.42390800	0.56901900
H	-3.02242100	0.41070000	0.90426900
H	-3.02009700	0.95079000	-0.54527900
H	-1.31787900	0.91574100	-2.03421300
H	-1.20032100	2.27387400	-1.30189500
H	3.30006300	-0.21155800	1.32352700
H	1.91317900	-0.26518800	0.72567300
H	2.69933300	1.46757800	-0.37968500
H	2.13616100	1.09335800	-1.74286100
H	0.94270800	2.91795800	-0.78163700
H	0.10706100	2.89239400	0.50970400
O	3.88654200	-2.07613300	0.22219800
O	0.31475400	2.00532300	2.17197200
O	-1.71600900	-0.01016400	2.39571800
O	-2.73442600	-2.12749200	-0.87436800
O	-1.00598400	-2.33541500	1.27655100
O	-3.53183800	0.38982600	0.07812100
O	-1.84422400	1.67270200	-1.73795800
O	2.51241800	0.33919000	1.20706600
O	2.52029800	1.84247900	-1.26116300
O	0.08740300	3.20671100	-0.41647200
O	-0.27724100	-0.94869000	-2.10666800
O	1.18151700	-1.53115900	-0.44849900
C	0.78290900	-0.97562000	-1.50091000

-953.4240 -953.15938 -953.1301 -953.2188

MD10-13

H	2.59651200	-1.31273400	2.72962800
H	2.84548500	-0.06855800	1.84301700
H	2.66763700	0.38661400	-2.04500800

H	4.19109600	0.32696800	-1.84503800
H	0.69875000	0.26878500	-0.99090400
H	0.52178500	1.77694400	-1.29590600
H	-0.86002400	1.43660600	2.08143000
H	-0.63315600	2.57903600	1.10212300
H	2.03316000	1.41439900	0.43275200
H	1.43386000	1.81915900	1.77002600
H	0.82624300	-1.84931800	-0.49270900
H	-0.70512200	-1.64671100	-0.98297700
H	-2.71026100	-0.82206200	-1.53663400
H	-2.93690600	-2.13470200	-0.77301300
H	2.85198700	-1.93188300	0.40501900
H	2.97408800	-1.72082300	-1.12041300
H	-1.01510500	-0.61867300	0.97338300
H	-2.21860200	0.29482200	0.73538800
H	-2.87681000	-1.68300800	1.41334300
H	-2.92994300	-3.21967500	1.55425900
O	3.14962200	-0.99729300	2.00929100
O	3.45674900	-0.16913400	-2.21880100
O	1.20210400	1.08118700	-1.22915100
O	-0.36595300	2.27009600	1.98915600
O	2.27431100	1.50270600	1.37563800
O	0.02290300	-1.28403200	-0.43521400
O	-2.48329600	-1.76893100	-1.55268300
O	2.57212800	-2.30754600	-0.45393600
O	-1.77867200	-0.22947500	1.44662600
O	-3.38441900	-2.48073200	1.13990000
O	-2.76484200	1.04834600	-0.81074500
O	-1.15008300	2.65689100	-0.82281700
C	-2.12651400	2.02135900	-1.24264700

-953.4243 -953.15878 -953.1301 -953.2176

MD10-14

H	-2.63191600	0.77498500	1.76178600
H	-3.62494400	0.98010100	0.58984000
H	-4.61752900	0.58736000	-1.56711800
H	-3.89834300	-0.67980400	-1.06432700
H	-0.33227300	-1.28423000	-0.10273100
H	0.29892900	-2.38249100	0.81427600
H	2.58071800	-2.20973100	0.91445300
H	2.33918700	-3.71902200	0.69825500
H	-2.23429200	-2.26736900	-0.77750100
H	-1.84549500	-1.23340600	-1.80811100
H	-0.55059400	0.73617500	-1.25486900
H	0.53779600	-0.15263600	-1.98046600
H	2.74444600	-0.66570200	-1.73464700
H	2.61623500	0.78811500	-2.04076400
H	-1.68602300	2.08469800	0.09137300
H	-0.24192600	2.67464100	-0.10379100
H	-0.96741000	-0.29012900	3.21033400
H	-1.29220900	-1.12354200	1.95430500
H	2.08819700	2.64836800	0.48307500

H	1.36403600	3.25825800	1.69450300
O	-3.01812500	1.46059300	1.18361700
O	-4.53887500	0.01913200	-0.79517200
O	-0.54782200	-1.94521200	0.59154900
O	2.01095500	-2.96445700	1.19515800
O	-2.59640600	-1.80732600	-1.54949800
O	-0.34921800	-0.18298600	-1.56599000
O	2.35086800	-0.07266000	-2.40144200
O	-0.99112500	2.26833100	-0.57733400
O	-1.70699600	-0.59110800	2.67468100
O	1.37243200	3.27252300	0.73329100
O	3.26150100	1.38556500	-0.01423900
O	3.40618100	-0.87743700	0.13738100
C	3.35440900	0.29261400	0.55435300

-953.4222 -953.15865 -953.1288 -953.2213

MD10-15

H	3.34401700	0.66099900	-0.81932100
H	1.93778900	0.85073200	-1.41646300
H	1.17492400	1.72516500	1.55717000
H	2.02885900	2.24397100	0.39101200
H	4.65667100	-0.68372900	0.79439600
H	3.52568600	-1.45535900	0.08377400
H	-1.20998800	-2.87738400	-0.44192100
H	-0.01219700	-3.30260000	0.41208300
H	1.43778700	-1.76821600	-0.49110900
H	1.48426900	-1.56765300	1.00213300
H	0.23122300	0.03467900	0.58716500
H	-0.43287500	-0.42258800	1.90235500
H	-1.70765600	-2.13725300	1.69409100
H	-2.58721400	-0.87788800	1.56758200
H	-1.06037400	4.28654100	1.21047000
H	0.13728400	3.46235400	0.67113700
H	-1.81336300	2.78705100	-0.52405100
H	-1.82770900	1.69175700	-1.62010000
H	-3.25384800	0.71725400	-0.13728500
H	-3.14320500	-0.72633400	-0.60361800
O	2.70157400	1.34742700	-1.07452700
O	1.52400500	2.55090400	1.17532100
O	4.30315400	-0.87687500	-0.07884400
O	-0.97754900	-3.37493800	0.36513700
O	1.84426200	-2.15882100	0.31008700
O	0.41300200	-0.05959400	1.53635400
O	-1.93322600	-1.30847100	2.15806600
O	-0.69916400	3.91827200	0.39954600
O	-2.40136800	2.16555700	-1.00173400
O	-3.52591100	-0.18977300	0.10795100
O	-1.55187100	-1.46118700	-1.76199500
O	0.46475100	-0.44628600	-1.45529600
C	-0.70055600	-0.58187000	-1.87667100

-953.4229	-953.15797	-953.1287	-953.2179

MD10-16

H	-1.03032700	2.33217700	0.93697600
H	-0.92317700	1.32769300	2.08922800
H	1.20450100	2.55280600	-1.97539100
H	0.27325400	3.59289400	-1.39452600
H	-0.84295500	1.50461800	-1.19088600
H	-2.31714500	1.60736900	-0.73422100
H	-2.25304900	-2.18542400	-2.08284900
H	-2.19574000	-2.35899100	-0.56346400
H	-3.01497400	0.05257600	0.71847500
H	-3.43113400	-0.50606700	-0.63852000
H	1.22938600	0.77912200	-0.65606500
H	0.83619200	-0.33565100	-1.67192700
H	0.37382100	-2.31953700	-0.79949500
H	1.82474600	-2.31691500	-1.32550400
H	1.03935600	2.08702600	1.32738100
H	1.96935100	2.61778500	0.20274700
H	2.42645200	0.19334000	1.42330900
H	1.54424100	-1.01940100	1.78764900
H	3.19364300	-1.75059100	0.36022100
H	4.19786600	-1.75430900	-0.81617200
O	-0.63396400	2.22006200	1.82545300
O	1.20732000	3.44914700	-1.60028400
O	-1.51136700	2.14653200	-0.88273500
O	-2.82124800	-2.21624900	-1.30593700
O	-3.48281800	0.30386900	-0.09755200
O	0.82301800	0.63891000	-1.52897700
O	0.90571100	-2.12799800	-1.59484800
O	1.90138400	1.91764900	0.87207700
O	2.47168000	-0.75821300	1.63450700
O	3.49619600	-2.27680400	-0.41775600
O	-0.82217100	-2.27476700	0.65854600
O	-1.50222500	-0.51887200	1.94987500
C	-0.72607200	-1.35444100	1.48401600

-953.4232	-953.15780	-953.1289	-953.2170

MD10-17

H	0.90127500	-0.45484000	-2.29850900
H	2.30632900	-0.35784200	-1.63708000
H	3.11217600	2.06790000	0.37921400
H	3.91499100	0.78442300	0.25643200
H	1.14544900	-1.18688500	1.84548000
H	0.70509000	-2.37736000	0.95038100
H	-1.61720400	-2.27687100	0.13341800
H	-1.29323600	-3.80093100	0.19532200
H	2.70197400	-1.50526000	0.38382500
H	4.07951300	-1.79524400	-0.30308100
H	0.73753500	0.97539700	1.57008500
H	-0.39234100	0.45841300	2.50760600
H	-2.54979400	-0.09457500	1.86467500

H	-2.63388600	1.37254500	2.26561000
H	1.20408800	1.33180000	-0.76743200
H	0.31307700	2.49036200	-0.24711600
H	-1.37131500	-1.03338300	-2.29610200
H	-0.56956300	-2.23124600	-1.81715800
H	-1.98466100	2.48196900	-0.88191600
H	-1.23483200	3.34028600	-1.91187300
O	1.62186100	0.16870900	-2.08576800
O	4.02005700	1.73177600	0.43133200
O	1.47683800	-2.01477300	1.41759000
O	-0.90169800	-2.94793100	-0.01274900
O	3.37767200	-1.14109100	-0.25290200
O	0.56461100	0.39109500	2.33706800
O	-2.31865200	0.52183800	2.58960100
O	1.07060300	1.93083100	0.00183000
O	-0.60198200	-1.57841800	-2.53746700
O	-1.33309900	3.20708800	-0.96447400
O	-3.10431700	1.04649000	-0.69963500
O	-2.73124700	-0.99071800	0.24552800
C	-2.76663900	-0.13130600	-0.65856900

-953.4201 -953.15698 -953.1270 -953.2182

MD10-18

H	-2.02452500	1.87458000	0.48863800
H	-1.24538500	1.12843100	1.59428200
H	-0.02194700	4.51150900	0.41897700
H	-0.28625200	3.13250500	1.06626400
H	-1.80667400	1.02325500	-1.55593500
H	-3.29628900	0.82381200	-1.17918100
H	-2.02618000	-1.70556400	0.87420000
H	-2.36361600	-2.95062400	0.03156900
H	-5.16996600	-0.31682000	-0.11949100
H	-3.93198900	-1.21151900	-0.33519000
H	-0.27179800	-0.11746600	-0.42803000
H	0.32929800	-0.72783100	-1.69363100
H	0.90809200	-2.62469600	-0.70677400
H	2.14300400	-2.19973600	-1.44382500
H	1.19857700	1.47339700	-1.26596000
H	1.41716400	2.77028700	-0.44900300
H	3.28445700	1.41042200	-0.42013300
H	4.03119300	1.22634000	0.91822100
H	3.81556800	-0.88966500	-0.16702500
H	3.00374800	-1.99871300	0.53318700
O	-1.58229700	2.01018400	1.35510000
O	0.43563000	3.75342700	0.79334400
O	-2.58852000	1.49398600	-1.21661900
O	-2.77863400	-2.23892000	0.52880500
O	-4.52584000	-0.57007200	-0.78692200
O	-0.09548300	0.09320100	-1.36437000
O	1.19774700	-2.39253300	-1.60539600
O	1.86370600	2.16244100	-1.07122600
O	4.05057700	0.94323100	-0.00067800

O	3.60120700	-1.84373900	-0.21368600
O	1.03218900	-2.27848300	1.34120100
O	-0.62048800	-0.71663100	1.38130000
C	0.39919100	-1.32677200	1.78533300

-953.4206 -953.15681 -953.1270 -953.2191

MD10-19

H	1.15613400	-2.26096100	1.38764800
H	2.63460900	-1.78369700	1.08446400
H	4.94441500	-0.92379100	0.83657000
H	3.81842000	-0.07718700	0.19597300
H	-0.98324100	0.81892800	0.56612000
H	-2.11416600	0.78818800	1.60335400
H	-3.98698100	-0.67229800	1.42655600
H	-3.70285200	0.17010000	0.17140400
H	-0.18313800	2.38756400	1.82099300
H	-0.27599900	3.91700500	1.68127500
H	2.60477200	-0.11912600	-1.65339700
H	2.67459200	1.35309600	-1.19186400
H	0.57408300	2.20050500	-1.24597700
H	1.19486500	2.89078900	-0.02561200
H	1.32167100	-1.73913500	-0.74306700
H	0.44098500	-0.61196400	-1.39558200
H	-1.06597500	-2.26110500	1.08408000
H	-0.74002000	-1.02540700	1.94001000
H	-2.19675800	-1.80075100	-1.05426000
H	-0.80035600	-2.41090600	-1.21082000
O	1.92029400	-2.37808100	0.78901800
O	4.07929500	-0.55001700	1.02477500
O	-1.14148500	0.85682000	1.53479500
O	-3.88222100	0.23847700	1.13252800
O	0.36113600	3.19794700	1.72111500
O	3.22736100	0.55246900	-1.32376200
O	1.38703900	2.64496200	-0.94873400
O	0.98407800	-1.38938500	-1.60261400
O	-0.63356000	-1.99291400	1.91822500
O	-1.64352300	-2.53577900	-0.74135800
O	-0.87941400	0.91402500	-1.26243500
O	-2.96181800	0.04323700	-1.53116300
C	-1.97542100	0.72434600	-1.82683000

-953.4211 -953.15638 -953.1270 -953.2164

MD10-20

H	2.72205600	0.13333900	1.67814600
H	3.20377200	-0.03174400	0.24045700
H	-0.01074300	-2.19052500	-1.66706900
H	1.42939300	-1.72333000	-1.98790400
H	-2.40412400	-1.05433000	-1.30948400
H	-1.08654800	-0.39030600	-0.82690500
H	2.20347100	2.91525300	-0.45251400
H	3.34594900	2.10683200	0.16817700

H	1.48710700	0.41576400	-1.18083300
H	2.81796000	0.78620900	-1.82980700
H	-4.89834600	-0.79883100	-1.15794600
H	-3.97479000	0.31221600	-0.59217300
H	-2.79080000	1.80264600	0.64452000
H	-3.02356300	0.45640800	1.37528100
H	2.65531000	-2.99421200	0.79131200
H	1.41924700	-2.92072200	-0.13969500
H	1.05828600	-1.46477700	1.61610600
H	0.53549100	-0.05012600	1.23282700
H	-1.20098000	-0.95221000	2.23987300
H	-1.92876200	-1.41867700	0.98302900
O	3.48029700	0.32800200	1.10102500
O	0.90478100	-2.51483100	-1.77991500
O	-1.50530300	-1.26131800	-0.98488800
O	3.14497800	2.71428700	-0.56630400
O	2.29919600	0.01285300	-1.56683700
O	-4.12111600	-0.27717300	-1.37591800
O	-3.48202500	1.14529100	0.85164700
O	1.69349000	-2.99342200	0.80660600
O	0.80279400	-0.59641900	1.99317300
O	-2.09571900	-1.10873400	1.89113800
O	-1.21358400	2.80989400	0.13178200
O	0.05664400	0.97593600	-0.34013200
C	-0.21247500	2.19769800	-0.18606700

-953.4202	-953.15536	-953.1262	-953.2149

MD10-21

H	1.57952200	2.55442500	-0.89574000
H	2.26892100	1.98721100	0.34852000
H	-0.13329200	-0.39766000	2.68162700
H	1.17368100	0.39367900	2.56235400
H	-1.68063300	-1.89634100	1.67665500
H	-0.25062400	-2.06457700	1.09915000
H	2.80437600	-2.19285200	-0.39595600
H	4.16443400	-2.11928500	-1.09734500
H	1.83947800	-0.32809900	0.47292500
H	3.29152000	-0.33133700	0.89659700
H	-3.75284000	-0.76958700	1.74480700
H	-3.38073500	-1.13780800	0.29076500
H	-2.39247500	-0.92416600	-1.71031600
H	-2.76128700	0.47570300	-1.16941300
H	0.24295600	2.90017300	1.01592100
H	-0.42544500	1.92360700	2.00013800
H	0.63567200	0.63124000	-2.17020600
H	0.68867300	1.66038600	-3.32972000
H	-1.15849300	2.05433300	-1.51921300
H	-1.60511500	2.28142500	-0.06653100
O	1.90740800	2.81381000	-0.02027900
O	0.28210700	0.45325500	2.93846300
O	-0.73909000	-1.97376900	1.93228400
O	3.76456200	-2.16793500	-0.22437600

O	2.48426100	0.19494500	0.99099600
O	-3.44016600	-1.50399100	1.20819200
O	-3.11398100	-0.43600200	-1.28368000
O	-0.62129700	2.68186600	1.41153800
O	0.61945600	1.59996800	-2.37148400
O	-1.95298400	2.07457400	-0.96370600
O	-0.57662900	-1.70572700	-2.21779400
O	0.97140400	-1.65212700	-0.53339300
C	0.36188500	-1.28857000	-1.56151900

-953.4185 -953.15530 -953.1253 -953.2164

MD10-22

H	-2.96191200	-1.59847900	-0.09540800
H	-1.83452100	-2.64599900	-0.31886100
H	-0.25235200	-1.86365100	0.92178000
H	0.56099600	-2.50312300	-0.24615000
H	-0.42497800	0.46029100	1.35132800
H	0.02886000	-0.47222100	2.50059400
H	3.23022500	1.54731400	1.10635500
H	1.87811500	1.10276100	0.48419300
H	2.31572700	-0.69151900	2.04057200
H	1.54611200	-2.02514000	2.02989900
H	0.51769500	-1.34428700	-2.18091700
H	2.03201400	-1.51398600	-1.88518000
H	3.48068400	0.37512200	-2.31529400
H	3.38838600	0.04338000	-0.81611400
H	-1.54099300	-0.80137200	-1.59818100
H	-0.56520800	0.38668700	-1.27299600
H	-3.42712300	0.53074600	0.50511700
H	-2.67262100	-0.23160700	1.61183200
H	-2.22993200	2.42634700	-0.52472600
H	-2.49649200	1.29633000	-1.50843600
O	-2.49224700	-2.11105800	-0.78839900
O	0.14631900	-2.69963100	0.62206200
O	-0.73847700	-0.25260300	1.94119800
O	2.78276200	0.77410400	0.74617100
O	1.83932500	-1.30966600	2.62019500
O	1.18500900	-1.99551000	-1.89619500
O	3.55055200	-0.35725700	-1.69538500
O	-0.99650400	-0.08941800	-2.00524400
O	-3.48090100	-0.26187400	1.07274300
O	-2.94462300	1.85213300	-0.84826800
O	0.30583400	1.53467900	0.05176400
O	0.72587200	3.77269400	0.19313800
C	0.06066000	2.77827700	0.01672200

-953.4200 -953.15468 -953.1257 -953.2139

MD10-23

H	3.25856200	0.01055200	-0.52741500
H	3.29978900	-1.46678700	-0.11015800
H	1.77855200	-2.51974100	-1.64000000

H	1.46821300	-2.99419200	-0.23642700
H	0.06001200	-1.20322000	-0.74868500
H	-0.73011700	-0.40663300	-1.82749500
H	-2.23864000	1.26359000	-1.17495800
H	-2.65676300	1.07103000	-2.66487900
H	-3.62028400	-2.20989500	-0.95144100
H	-3.52271100	-0.68429000	-1.11869200
H	0.67799300	-1.58988200	1.45006500
H	-0.78518200	-2.03715900	1.20837600
H	-1.95342900	-0.29980700	1.84730800
H	-3.00094500	-1.17466100	1.10243800
H	2.35578000	-0.26935500	1.59832000
H	1.01365300	0.38866600	2.10235200
H	2.08227300	1.82786500	-1.33476300
H	1.51177700	0.44964100	-1.78270000
H	0.83023300	3.12052900	0.23131600
H	2.30799900	3.16491000	0.64491700
O	3.54644800	-0.57870100	0.20199300
O	2.18256700	-2.98969800	-0.89894200
O	0.12720000	-0.82907700	-1.65404000
O	-2.44185800	0.57768500	-1.86753300
O	-3.96329500	-1.38440000	-0.59568300
O	0.12198400	-2.14600900	0.85345900
O	-2.33002300	-1.19848700	1.80673700
O	1.63776000	-0.33803500	2.26319200
O	2.32361000	0.99371600	-1.77528800
O	1.73203100	3.30357300	-0.11450300
O	-0.75399400	1.28257400	1.68933800
O	-1.76388700	2.39165900	-0.03720100
C	-0.91418500	2.14673800	0.82671400

-953.4183 -953.15455 -953.1251 -953.2151

MD10-24

H	0.87429300	-1.57862400	-1.47009000
H	1.47663900	-1.25388700	-0.08772100
H	2.44169200	1.25237700	2.67048500
H	2.49789400	-0.16371200	2.10459500
H	-0.46700300	1.60721700	1.47982800
H	-1.54909700	0.49056600	1.61603100
H	-1.83087200	-3.60259500	1.56565800
H	-1.88005300	-2.15501000	0.97810700
H	0.33626900	-0.47205700	1.83617700
H	0.39531700	-2.01801700	1.73644800
H	0.88902500	2.53917400	-0.08457800
H	-0.60032700	3.01277700	-0.26507700
H	-2.62736700	2.18725800	-1.25103900
H	-2.91395600	3.56127800	-0.63487400
H	2.03302600	0.70138500	-0.91390700
H	2.76765900	1.31470100	0.28385600
H	-1.50591700	-1.93645900	-1.77291000
H	-0.85888300	-2.94516300	-0.83973800
H	3.45953200	-1.00547900	-1.86978100

H	4.12830900	0.34624600	-1.89983900
O	1.64830200	-1.15857700	-1.04653000
O	2.99522500	0.67427200	2.13461300
O	-0.74451100	0.84225000	2.03684500
O	-1.29078400	-2.94460900	1.11938700
O	0.96439700	-1.23182600	1.70985000
O	0.11010000	2.89018500	0.39171100
O	-2.26805700	3.09583600	-1.17432600
O	2.41424300	1.54243700	-0.59596100
O	-0.75259600	-2.54948300	-1.72140900
O	4.26837500	-0.56623400	-2.18090500
O	-3.32607500	0.50252100	-1.22088100
O	-2.69763300	-0.72350900	0.60002100
C	-2.93901300	-0.47801300	-0.60015900

-953.4176 -953.15445 -953.1244 -953.2164

MD10-25

H	-1.59186700	1.81499700	-0.91725200
H	-2.66424800	0.67899800	-1.29903500
H	-4.22565800	-1.07262100	-2.30229100
H	-3.83716300	-1.11315600	-0.80384300
H	-2.15173300	0.60361200	0.99324200
H	-1.39483500	0.37563600	2.30243600
H	0.84509900	1.20678000	1.97205400
H	0.76264100	-0.25681100	2.44987200
H	-2.78797000	-1.38208500	1.14365800
H	-2.10496000	-2.21143400	0.05229800
H	0.57799400	-1.85319700	0.95023600
H	2.04994300	-1.92033200	1.39384600
H	3.41244600	-2.02349000	-0.50770700
H	3.54063100	-0.69386700	0.20231100
H	-0.71605400	-0.64114800	-0.82172300
H	0.39731300	-1.58444900	-1.36063700
H	0.03570900	3.13116600	-0.21874300
H	-1.24843000	3.73069300	0.38313800
H	2.38287700	-0.47208200	-1.88505800
H	2.17894600	-1.25198800	-3.21492300
O	-1.80696600	0.86102100	-0.87416700
O	-4.16391800	-0.51529700	-1.52183200
O	-2.27790400	0.25571000	1.89397100
O	0.42919000	0.64458300	2.64624500
O	-2.97890200	-2.04030700	0.43975400
O	1.14036100	-1.90859100	1.74467200
O	3.64746100	-1.65206600	0.35287300
O	-0.34972200	-1.54471200	-0.73811500
O	-0.86813500	3.44653300	-0.45389700
O	2.10344900	-1.34154100	-2.26092000
O	2.87549100	0.82076700	-0.82347800
O	1.51213600	2.27210400	0.29133800
C	2.07316500	1.75819100	-0.68057100

-953.4184 -953.15433 -953.1248 -953.2147

MD10-26

H	1.21991400	1.17011600	1.50786600
H	1.08405700	1.68185200	0.06499400
H	-1.52359100	-0.65475000	1.89812700
H	-1.36934000	0.10032800	0.58377300
H	2.19952700	-0.92564600	3.06373100
H	2.61198900	-0.62922700	1.59600800
H	2.55505200	0.39664000	-2.20144000
H	2.62917300	-1.09261500	-2.11647000
H	3.31528200	1.08588400	0.63668800
H	3.72381700	-0.01527200	-0.36086000
H	0.21230100	-1.97975600	1.95749200
H	-1.01173200	-2.60944500	1.21709200
H	-0.93494700	-2.49518300	-1.05180900
H	-2.39591700	-2.16775100	-0.71705000
H	-0.49709800	2.91539800	1.21835100
H	-1.70214300	2.05981900	1.57564100
H	-2.08127700	2.48691000	-0.59784500
H	-1.31761600	1.77231700	-1.70880700
H	-3.25486200	0.27226500	-1.19042500
H	-3.40901600	-0.33962100	0.18135200
O	1.35772400	1.96043400	0.96176300
O	-1.83539900	0.15520100	1.43450600
O	1.83871500	-0.82040400	2.17843400
O	3.21303100	-0.31647600	-2.12066700
O	3.79168000	0.24191200	0.58391800
O	-0.70669700	-2.26707300	2.08396200
O	-1.69268200	-2.81707900	-0.53157000
O	-1.46785900	2.93928600	1.22748400
O	-2.21619400	2.02011100	-1.44449600
O	-3.60698400	-0.52324200	-0.75029300
O	0.52557800	-1.39160700	-1.71251000
O	0.65000800	0.88412200	-1.57267400
C	0.18984300	-0.25895700	-1.37654300

-953.4137 -953.14929 -953.1196 -953.2091

MD10-27

H	1.16186600	1.70321800	1.17086600
H	2.34725900	1.60153700	0.17364000
H	1.99458600	-1.69344000	-2.17538300
H	2.87920500	-0.43696600	-2.11041100
H	-0.17008900	-0.10272500	-1.55071600
H	-0.37391300	1.15645300	-0.67662500
H	-2.19520400	2.18923600	-1.62011200
H	-3.15118600	1.73256900	-0.52112600
H	1.41480100	1.32455100	-1.84450200
H	2.60658400	2.16395200	-2.43992400
H	0.57173300	-1.86761300	-0.37928200
H	-0.81682800	-2.20105300	-1.04430300
H	-3.01761300	-1.70607100	-0.76971700

H	-2.59188200	-2.58012000	0.36346900
H	2.03409100	-0.54046900	0.89808100
H	2.67452500	-1.54822500	-0.07494800
H	-1.31835000	1.31624300	1.47968600
H	-1.39673200	2.48970300	0.50118000
H	3.57397600	-0.86797600	2.52030600
H	3.39437900	0.63071800	2.47607500
O	2.07450700	1.37312500	1.07850000
O	2.91149900	-1.40664800	-2.04636000
O	-0.17263100	0.88366500	-1.59275600
O	-2.72609600	2.53937500	-0.89325000
O	2.40291000	1.46138700	-1.81618000
O	0.07652000	-1.82894500	-1.22125900
O	-2.55216300	-2.54572700	-0.60805600
O	2.13324900	-1.49710600	0.73382300
O	-0.75037800	1.96992400	1.02265200
O	3.91483600	-0.05655300	2.91824900
O	-2.83519800	-1.21160500	1.95143200
O	-3.45300100	0.10898800	0.19512100
C	-2.96893900	-0.18042700	1.30807000

-953.4117	-953.14772	-953.1182	-953.2089

S-7 References

(1) Ma, L., Majer, K., Chirot, F., Issendorff, B. von. Low temperature photoelectron spectra of water cluster anions. *J. Chem. Phys.* **2009**, *131*, 144303.

(2) Höckendorf, R. F., Balaj, O. P., van der Linde, C., Beyer, M. K. Thermochemistry from Ion-Molecule Reactions of Hydrated Ions in the Gas Phase: A New Variant of Nanocalorimetry Reveals Product Energy Partitioning. *Phys. Chem. Chem. Phys.* **2010**, *12*, 3772–3779.

(3) Akhgarnusch, A., Tang, W. K., Zhang, H., Siu, C.-K., Beyer, M. K. Charge Transfer Reactions between Gas-Phase Hydrated Electrons, Molecular Oxygen and Carbon Dioxide at Temperatures of 80-300 K. *Phys. Chem. Chem. Phys.* **2016**, *18*, 23528–23537.

(4) Berg, C., Schindler, T., Niedner-Schatteburg, G., Bondybey, V. E. Reactions of Simple Hydrocarbons with Nb_n^+: Chemisorption and Physisorption on Ionized Niobium Clusters. *J. Chem. Phys.* **1995**, *102*, 4870–4884.

(5) Bondybey, V. E., English, J. H. Laser Induced Fluorescence of Metal Clusters Produced by Laser Vaporization: Gas Phase Spectrum of Pb_2. *J. Chem. Phys.* **1981**, *74*, 6978–6979.

(6) Dietz, T. G., Duncan, M. A., Powers, D. E., Smalley, R. E. Laser Production of Supersonic Metal Cluster Beams. *J. Chem. Phys.* **1981**, *74*, 6511–6512.

(7) Maruyama, S., Anderson, L. R., Smalley, R. E. Direct Injection Supersonic Cluster Beam Source for FT-ICR Studies of Clusters. *Rev. Sci. Instrum.* **1990**, *61*, 3686–3693.

(8) Bersenkowitsch, N. K., Ončák, M., Heller, J., van der Linde, C., Beyer, M. K. Photodissociation of Sodium Iodide Clusters Doped with Small Hydrocarbons. Chem. Eur. J. **2018**, 24, 12433–12443.

(9) Heller, J., Ončák, M., Bersenkowitsch, N. K., van der Linde, C., Beyer, M. K. Infrared Multiple Photon Dissociation of Cesium Iodide Clusters Doped with Mono-, Di- and Triglycine. Eur. J. Mass Spectrom. **2019**, 25, 122-132.
